# Supramolecular Gels as Active Tools for Reaction Engineering

**DOI:** 10.1002/anie.202502053

**Published:** 2025-04-07

**Authors:** David K. Smith

**Affiliations:** ^1^ Department of Chemistry University of York Heslington York YO10 5DD UK

**Keywords:** Catalysis, Gels, Self‐assembly, Supramolecular, Synthesis

## Abstract

Supramolecular gels, assembled from low‐molecular‐weight gelators (LMWGs), are fascinating soft materials for use in synthesis, combining aspects of hetero‐ and homogeneous systems. The unique combination of environments within a gel offers the ability to control reactivity in new ways. For example, self‐assembly into a gel network can modify the reactivity of catalytic sites on the LMWG. Controlling the assembly of multiple LMWGs can result in integrated gels with orthogonal activities that could not normally coexist. Enzymes encapsulated within self‐assembled gels can exhibit superactivity, extending their use into solvent media more appropriate for organic synthesis. Highly reactive species, such as ligand‐free nanoparticles or moisture/air‐sensitive organometallics, can be protected within the unique environment of a supramolecular gel, facilitating their use in ambient conditions, potentially opening up the use of such species to nonspecialist researchers. Beyond fundamental chemistry, performing reactions in gels leads to the emerging concept of gels as “nanoreactors”. Smart chemical engineering methods are enabling the fabrication of materials and devices for use in a variety of synthetic workflows, potentially transforming the way synthesis is done. In summary, this review provides an overview of key concepts and signposts the way toward future developments of gels as active tools for reaction engineering.

## Graphical abstract

The unique combination of environments within gels, self‐assembled from low‐molecular‐weight gelators, offers the ability to control reactivity in new ways, achieving unique reaction outcomes, leading to the emerging concept of supramolecular gels as nanoreactors. Smart chemical engineering methods enable the use of such gels to fabricate materials and devices potentially capable of transforming the way synthesis is done.

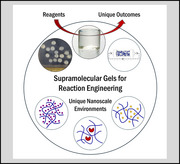

## 1 Introduction to Supramolecular Gels

**Figure 1:**
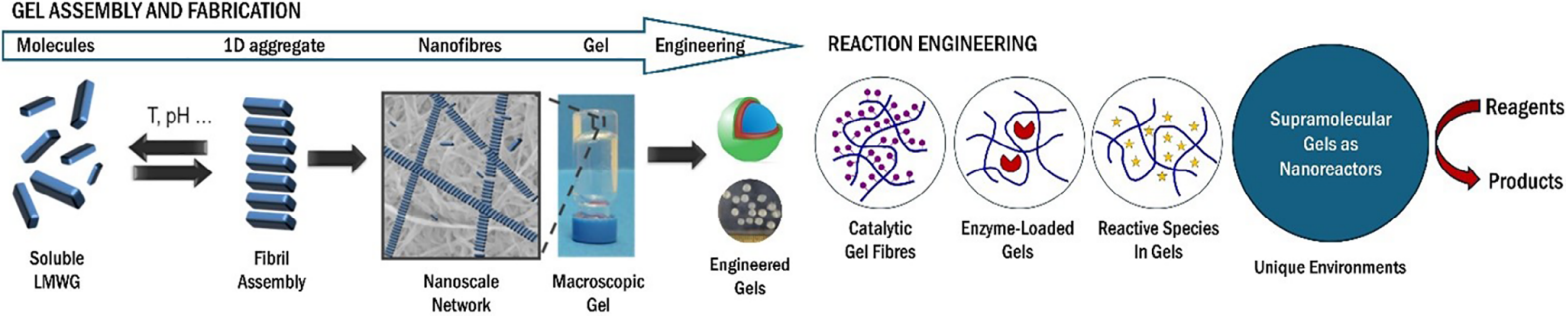
Schematic of supramolecular gel assembly from low‐molecular‐weight gelators to create engineered gel‐phase materials adapted from Reference [6] with permission from the Royal Society of Chemistry, and a schematic of the ways gels can be applied in reaction engineering with catalytic gel fibers, enzyme‐loaded gels, or reactive species in gels, all of which can make use of the unique nanoscale environment within gels to achieve unique reaction outcomes.

Supramolecular gels are fascinating soft materials in which molecular‐scale building blocks assemble into gels as a result of noncovalent interactions. Of particular interest are materials based on low‐molecular‐weight gelators (LMWGs) that self‐assemble into a sample‐spanning “solid‐like” nanoscale network within a mobile “liquid‐like” phase (Figure 1).^[^ ^1^, ^2^, ^3^, ^4^ ^]^ These colloidal systems combine the properties of two different phases in a single integrated material. In contrast to more widely used polymer gels, supramolecular systems have enhanced reversibility, with the potential to be disassembled into molecular‐scale building blocks.^[^ ^5^ ^]^ They also have remarkable synthetic molecular‐scale tunability.

Self‐assembly of LMWGs (Figure 1) is driven by directional noncovalent interactions, typically they assemble into 1D nanoscale fibrils. These fibrils then often bundle into larger fibers via lateral fibril–fibril interactions to form well‐defined nanofibers. These fibers form a sample‐spanning network in which fiber–fiber interactions provide the resulting gel‐phase materials properties. Gels that assemble in water are referred to as hydrogels—the hydrophobic effect often plays a key role in underpinning assembly. Gels formed in organic solvents are described as organogels—electrostatic interactions, like hydrogen bonds and π−π stacking, often help drive their assembly. Gels in ionic liquids are called ionogels, and those in deep eutectic solvents are known as eutectogels. For an LMWG to form a gel in a given solvent, it must be sufficiently soluble to dissolve and reorganize itself on the molecular‐scale but sufficiently insoluble to subsequently self‐assemble into a solid‐like network. If an LMWG is too soluble, it simply dissolves, if it is too insoluble, it simply precipitates. This balanced solubility profile is an essential factor in all supramolecular gels.^[^ ^7^ ^]^ Many LMWGs have a degree of amphiphile character encouraging self‐assembly, which is then reinforced by more directional local interactions.

Supramolecular gels can be based on a vast array of molecular‐scale building blocks. Self‐assembling peptides are particularly well‐established.^[^ ^8^, ^9^, ^10^, ^11^ ^]^ Typically, such systems assemble as a result of amide–amide hydrogen bonds combined with interactions between hydrophobic surfaces. The ability to easily modify peptides at the individual amino acid level provides significant tunability. In addition to peptides, there has also been considerable focus on gels based on nucleobases and/or sugars, both of which are naturally occurring building blocks with hydrogen bonding potential that can be easily modified to help direct self‐assembly.^[^ ^12^, ^13^, ^14^, ^15^ ^]^ Within a supramolecular gel, the nanoscale network acts as a heterogeneous phase, but the nanostructuring maximizes its effective surface area. Furthermore, the solvent, which acts as the homogeneous phase must have a good degree of compatibility with the LMWG network to allow it to extend through the bulk liquid‐like medium. These properties, therefore, mean that gels have unique potential for use in solution‐phase synthetic chemistry. Diffusion in the mobile liquid‐like phase is often fast on the scale of small molecules,^[^ ^16^ ^]^ meaning reagents can easily diffuse into a gel and products can diffuse out. This provides gels with considerable potential for use in synthesis.^[^ ^17^ ^]^

One simple advantage of using gels is that they enable easy separation of a catalyst from the reaction medium,^[^ ^18^, ^19^ ^]^ facilitating product purification and catalyst reuse. However, this review is focused on examples in which the supramolecular gel plays an active role in manipulating reactivity, not just acting as a solid‐like phase. Nanoconfinement opens up a number of possible advantages of gel‐based materials as reaction media as will be exemplified through the article. In general, nanoconfinement can modify reactions by (for example) increasing local concentration, stabilizing interactions between catalyst and reagent, reorganizing molecules for reaction, altering product selectivity, optimizing mass transport, stabilizing reactive species, or directly assisting in bond formation/breaking.^[^ ^20^ ^]^

A significant number of review articles about catalysis in gels have been published over the years,^[^ ^21^, ^22^, ^23^, ^24^ ^]^ especially focusing on peptide‐based LMWGs as organocataysts.^[^ ^25^, ^26^, ^27^, ^28^, ^29^, ^30^ ^]^ However, this new article explores the wide range of synthetic chemistry that can be enabled using varied gels and focuses on cases where the supramolecular gel plays an active role in controlling reactivity to achieve unexpected, but desirable, synthetic outcomes. This article does not aim to be comprehensive, but instead provides a commentary on key conceptual advances in terms of actively controlling reactivity within gels. In each case, I explore the mechanisms through which supramolecular gels have their unique effects on reaction outcomes. I aim to demonstrate how gels can be engineered to act as unique nanoreactors, which may enable new approaches to reaction engineering, leading to new and innovative workflows that may ultimately help change the ways in which synthetic chemistry is done.

## 2 Supramolecular Gels as Active Catalysts

### 2.1 Enhanced Catalysis by Gel Assembly

**Figure 2:**
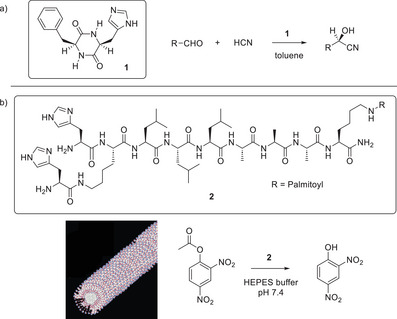
a) Structure of imidazole‐functionalized catalyst **1** reported by Inoue and coworkers, and the asymmetric aldehyde hydrocyanation reaction catalyzed by **1**. b) Structure of self‐assembling peptide amphiphile **2** reported by Stupp and coworkers, schematic diagram of its self‐assembly into nanofibers, and the hydrolysis of 2,4‐dinitropenyl acetate catalyzed by **2**. Schematic image adapted from Reference [35] with permission of the American Chemical Society.

Intriguingly, the earliest example of a unique reaction outcome using a self‐assembling gel was published before most academic scientists had recognized supramolecular gels as a distinct field of research. In 1990, as a part of very early interest in the field of organocatalysis, Inoue and coworkers investigated the addition of hydrogen cyanide to an aldehyde using an imidazole‐functionalized cyclodipeptide catalyst (**1**) (Figure 2a).^[^ ^31^ ^]^ The precise role of gel assembly is somewhat unclear as it was not the authors’ primary focus, but while performing the reaction between benzaldehyde and HCN in benzene, they found that the enantioselectivity was good at lower conversions, but became worse as conversion increased. They noted that the reaction changed over time from gel to solution. Furthermore, on changing the solvent to toluene, the gel remained stable throughout the reaction, and in this case, the enantioselectivity remained high throughout. The most effective reaction conditions were in toluene at low temperature (−20 °C). This work, therefore, provided the first hints that gel‐mediated organocatalyzed reactions may differ to those in solution. In the ensuing years, others worked further on this system and confirmed the importance of the self‐assembled gel‐phase.^[^ ^32^, ^33^ ^]^ Modelling studies indicated that the catalytically active species is a dimer, in agreement with the dependence of catalytic proficiency on self‐assembly.^[^ ^34^ ^]^

In 2007, Stupp and coworkers also made use of imidazole as a part of a self‐assembling peptide amphiphile (**2**), but in this case, they were deliberately exploring the impact of self‐assembly on organocatalysis (Figure 2b).^[^ ^35^ ^]^ Compound **2** formed gels above pH 6.5 but was fully soluble below pH 4, at which point the histidine is protonated. The researchers also created related compounds that self‐assembled into spherical micelles rather than cylindrical nanofibers. Their reaction of choice was the hydrolysis of 2,4‐dinitrophenyl acetate at pH 7.4, and it was demonstrated that the activity of the gel nanofibers was significantly greater than that of other assembled systems or, indeed, unfunctionalized imidazole in free solution. The researchers proposed that this rate enhancement was a result of the high‐density presentation of reactive sites on the surface of the self‐assembled nanofibers, emphasizing the high‐fidelity and degree of order associated with the β‐sheet assembly process.

Early work on gels also considered palladium‐based catalysis in the Suzuki–Miyaura cross‐coupling reaction between aryl bromides and aryl boronic acids and once again found that the assembled morphology affected catalytic performance.^[^ ^36^ ^]^ In this case, Pd–N coordination interactions mediated the binding of the metal ions and the formation of the gel, and gel morphology was dependent on the degree of palladium loading. The fibrillar network formed at higher Pd(II) loading levels had higher catalytic activity than the networks of spherical assemblies formed at lower loading. However, the reasons for this difference in activity were not fully elucidated. Also in early studies of metal‐based gels, Liu and coworkers reported a self‐assembled LMWG that bound Cu(II) to form multilayer gel‐phase nanotubes, which were more effective in the Diels–Alder reaction and gave a greater degree of enantioselectivity than equivalent catalytic copper complexes in solution or other nanostructures, such as fibers or flakes.^[^ ^37^ ^]^ It was suggested that the alignment of the catalytic sites on the surface of the nanotube and the resulting local stereochemically‐controlled environment played a role.

**Figure 3:**
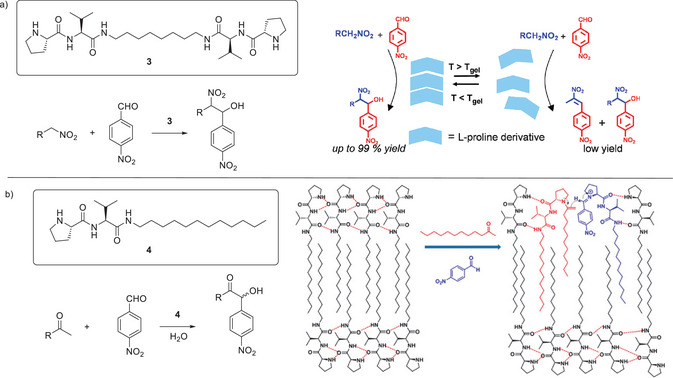
a) Structure of bola‐amphiphile proline‐functionalized LMWG **3** for catalysis of the nitroaldol reaction between nitroalkanes and 4‐nitrobenzaldehyde. Schematic of self‐assembly illustrating that when assembled in the gel phase, the reaction proceeds in high yield, whereas when disassembled, for example at elevated temperature, the reaction is less effective. Schematic reproduced from Reference [38] with permission of the American Chemical Society. b) Structure of proline‐functionalized LMWG **4** for catalysis of the aldol reaction between ketones and 4‐nitrobenzaldehyde. Schematic of self‐assembly illustrating how interactions between the alkyl chain on the aldehyde and the self‐assembled gel can raise the local concentration of the aldehyde. Schematic reproduced from Reference [43] with permission of the Royal Society of Chemistry.

Since these early studies, the concept of synthesizing LMWGs capable of acting as catalysts and then assembling them into nanostructured gels with modified or enhanced performance has become an important general strategy in organocatalysis.^[^ ^21^, ^22^, ^23^, ^24^, ^25^, ^26^, ^27^, ^28^, ^29^, ^30^ ^]^ At the forefront of this field, Escuder and coworkers have made very significant contributions in terms of developing a detailed understanding of the ways gel assembly can modify or enhance catalytic ability. In a key 2009 paper, these researchers reported a bola‐amphiphile gelator (**3**), functionalized with proline groups on the periphery, which formed gels in solvents such as nitromethane and nitroethane (Figure 3a).^[^ ^38^ ^]^ Compound **3** had modified basicity on self‐assembly into the gel, as indicated by the ability of the gel, but not the sol, to induce bromothymol blue to change color. As a result, the gel‐phase could catalyze the nitroaldol reaction between the weakly acidic solvent and 4‐nitrobenzaldehyde, whereas the individual LMWG molecules in solution could not. Catalytic activity could be switched on/off by varying the temperature below/above the gel–sol transition temperature. The researchers argued that bringing basic proline groups into close proximity on the gel nanofibers modifies their effective p*K*a value,^[^ ^39^ ^]^ hence driving catalysis. At a similar time, Ulijn and coworkers also noted that the p*K*a values of self‐assembled peptide gelators could, remarkably, be shifted by as much as 6 pH units as a result of self‐assembly.^[^ ^40^ ^]^ In a similar way, the p*K*a values of individual amino acids in enzymes are often dramatically changed as a result of their local microenvironment, underpinning their reactivity.^[^ ^41^, ^42^ ^]^ The ability of self‐assembly to organize molecular‐scale fragments on the nanoscale and change their surrounding microenvironment to promote reactivity is an easy way of enhancing the performance of synthetically simple building blocks.

Escuder and coworkers went on to report the use of a basic proline‐functionalized organogelator for the conjugate addition of cyclohexanone to nitrostyrene.^[^ ^44^ ^]^ Fascinatingly, the enantioselectivity of the reaction was inverted depending on whether the LMWG was in its self‐assembled gel state or free in solution. In this case, the authors proved that the changes were not due to enhanced basicity, but that the different hydrogen bonding pattern in the assembled gel state changed the steric environment of the catalytically active site, which was rigidified. This example demonstrated that not only could reaction rates be enhanced by self‐assembly but also different products could result (i.e., enhanced selectivity). Liu and coworkers also reported a supramolecular gel that exhibited enhanced enantioselectivity in the aldol reaction as a result of self‐assembly.^[^ ^45^ ^]^ Intriguingly, they reported that the local chirality of the proline did not solely control the outcome, but the overall helicity of the self‐assembled nanofibers appeared to amplify enantioselectivity. This importance of self‐assembled morphology was further emphasized by work on amyloid peptides in which two different peptides, (RF)_4_ and proline‐loaded P(RF)_4_, were combined and used to catalyze the aldol reaction between cyclohexanone and 4‐nitrobenzaldehyde.^[^ ^46^ ^]^ Self‐assembled (RF)_4_ had a degree of catalytic activity, but on increasing the loading of P(RF)_4_, the catalytic activity actually got worse even though more proline was present. Careful structural studies indicated the assemblies became more tightly packed, and it was assumed that the proline groups therefore became less accessible to the reagents.

In 2013, a proline‐based LMWG (**4**) was used to catalyze the aldol reaction between an aliphatic ketone and 4‐nitrobenzaldehyde (Figure 3b).^[^ ^43^ ^]^ There was a clear preference for more hydrophobic ketones, with the yield increasing with log*P* value. It was suggested these ketones accumulated within the hydrophobic interior of the self‐assembled gel phase and hence reacted more effectively with the catalyst. The reaction did not proceed with water‐soluble ketones, which were presumed to remain in the aqueous liquid‐like phase. This hydrophobically‐driven organocatalysis mechanism became an important concept for enhancing reactivity in supramolecular gels. For example, Alves and coworkers explored a family of lipopeptides that catalyze the aldol reaction between cyclohexanone and 4‐nitrobezaldehyde in water.^[^ ^47^ ^]^ Molecular dynamics methods were used to understand self‐assembly and packing in more detail, showing that the water content close to the proline group could influence the reaction outcome. It is also worth noting, in passing here, that catalytic peptide assemblies have moved far beyond just gel‐type materials and now explore a wide range of different assembled nanostructures.^[^ ^25^, ^26^, ^27^, ^28^, ^29^, ^30^ ^]^

Reflecting on the fact that biological systems can achieve very subtle control over reactivity profiles, Escuder and coworkers went on to determine whether gels could achieve similar outcomes. They investigated an LMWG capable of assembling into gels that exist in a variety of polymorphs, which have different self‐assembled structures depending on heating temperature, ageing time, use of ultrasound, and pH switching.^[^ ^48^ ^]^ The “history” of a gel can play a key role in controlling its self‐assembled structure and properties. The researchers used four different polymorphic gels to catalyze the aldol reaction between cyclohexanone and 4‐nitrobenzaldehyde, with different rates of reaction being observed in each case. It was proposed that differences in molecular‐scale packing between different polymorphs may mean catalytic sites have different accessibilities. In support of this argument, they noted that the tape‐like polymorph, with a lower aspect ratio and lower surface area, showed a lower reaction rate than fibrillar polymorphs that have a higher aspect ratio and might hence be expected to display the catalytic sites more effectively.

**Figure 4:**
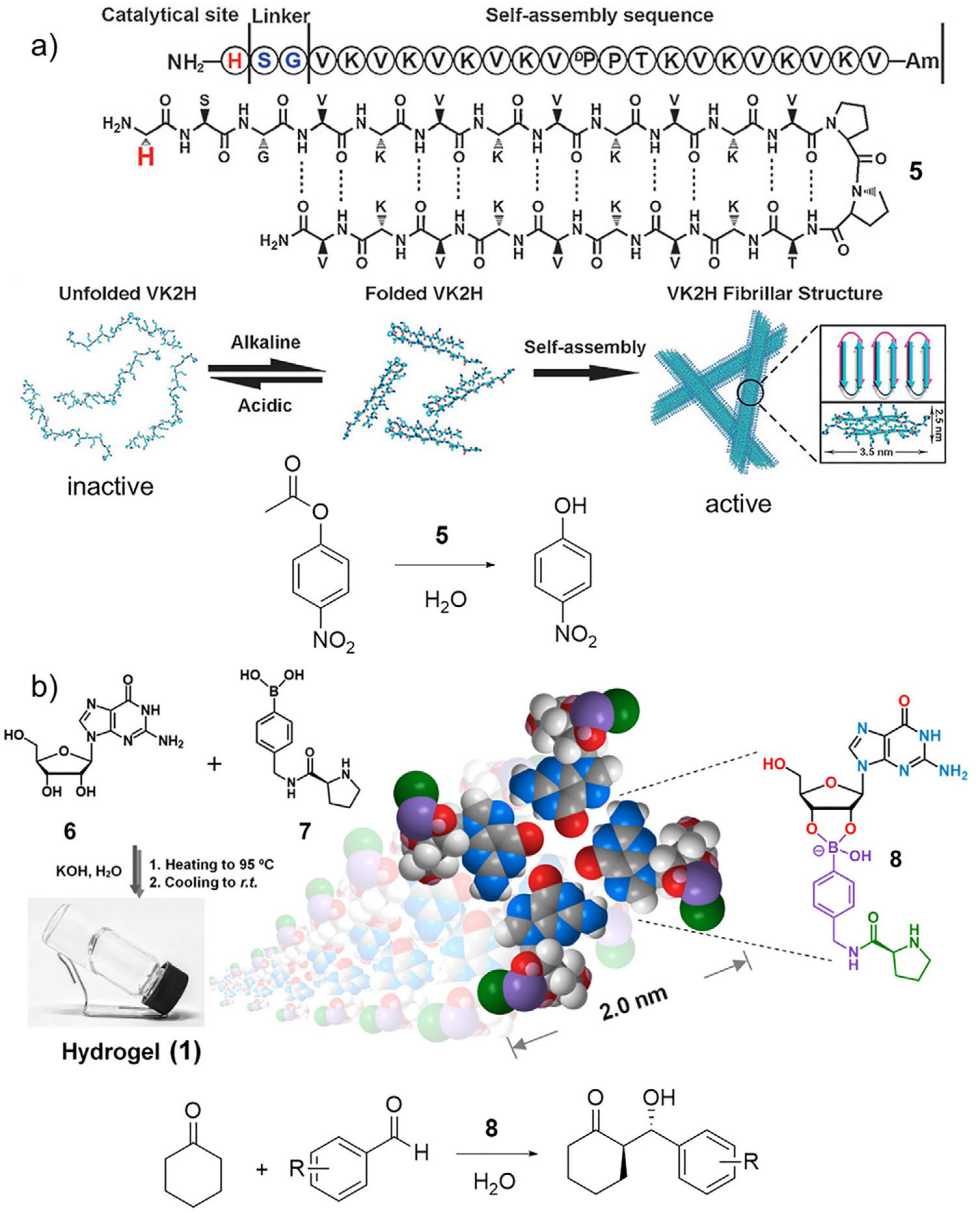
a) Peptide **5** folds and unfolds depending on the ambient pH, with the folded β‐sheet form assembling into fibrils and then gels that can catalyze the hydrolysis of 4‐nitrophenyl acetate. Scheme adapted from Reference [49] with permission of Wiley‐VCH. b) Compounds **6** and **7** react through dynamic boronic acid/diol chemistry to form compound **8** that assembles into G‐quartets through hydrogen bonding, which assemble into fibers and subsequently forms a gel. This gel catalyzes the aldol reaction between cyclohexanone and substituted benzaldehydes. Scheme adapted from Reference [51] with permission of the American Chemical Society.

In elegant work, Ulijn and coworkers reported a switchable gel‐forming system in which they could turn the hydrolytic activity of histidine amino acids on and off.^[^ ^49^ ^]^ They achieved this by creating a peptide (**5**) with a conformation that could be switched from random coil to β‐sheet dependent on pH (Figure 4a). On switching, the β‐sheet self‐assembles to form long fibrils with a hydrophobic edge and histidine residues extending in an ordered array. This constitutes a catalytic microenvironment that can bind a substrate and exhibit esterase‐like hydrolysis. At higher concentrations, these fibrils formed sample‐spanning gels that were catalytically active. As such, this system uses a variety of smart features, including self‐assembly, to optimize catalytic performance. Alternatively, with the goal of simplifying hydrolytic peptide design as far as possible, Marchesan and coworkers developed an unmodified tripeptide (His–Phe–Phe) that formed a thermoreversible gel capable of hydrolyzing 4‐nitrophenylacetate when assembled into supramolecular structures, but not as free molecules in solution.^[^ ^50^ ^]^

In addition to peptide‐based gels, other types of LMWG have also been of interest as organocatalysts. For example, Qiao, Li and coworkers reacted guanosine (**6**) with a proline‐modified boronic acid (**7)** via dynamic boronic acid mediated chemistry. The resulting proline‐modified guanosine (**8**) assembled into G quartets via base‐mediated hydrogen bonding, that then assembled further into fibrils and hydrogels (Figure 4b).^[^ ^51^ ^]^ The presence of proline meant the G‐quartet gels were active catalysts for the aldol reaction between cyclohexanone and benzaldehyde derivatives, with assembly enhancing catalysis and hydrophobic transition state stabilization being proposed. In fascinating work, D'Anna and coworkers demonstrated that even simple amino acids can be used as catalytic LMWGs in deep eutectic solvents.^[^ ^52^ ^]^ For example, l‐proline was an effective LMWG in these solvents and was catalytically active in the aldol reaction between propanone and 4‐nitrobenzaldehyde, performing more effectively than solution‐phase equivalents. Better results were obtained with more flexible substrates, suggesting such systems could better interact with the self‐assembled gel fibers. Using gels in deep eutectic solvents opens an alternative potentially eco‐friendly way of performing synthetic chemistry, avoiding the use of organic solvents.

Moving toward more complex synthetic processes, tandem reactions can also be promoted using supramolecular gels. Escuder and coworkers made use of a triazole‐functionalized LMWG that could bind copper(II) ions and was capable of catalyzing both “click” and aldol reactions within a single gel leading to the reaction of 1‐azido‐2‐propanone with both phenylacetylene (click) and 4‐nitrobenzaldehyde (aldol).^[^ ^53^ ^]^ In recent work, Ventura and coworkers created an amyloid peptide that assembled into a hydrogel and was also capable of showing two different types of catalytic activity.^[^ ^54^ ^]^ The peptide was designed with an alternating His–Tyr structure, and the self‐assembled nanofibers thus combined both hydrolase (His) and oxidase (Tyr) activities. The esterase‐type activity of His was demonstrated by hydrolysis of 4‐nitrophenyl acetate, whereas oxidase activity was achieved by electrochemical oxidation, mediated by Cu(II) binding to Tyr radicals, and leading to the oxidation of pyrrole to poly(pyrrole). This system, therefore, neatly incorporated two types of catalytic activity into a single self‐assembling motif. The ability to perform multiple reaction processes in one‐pot is an important theme in the development of telescoped synthetic processes that can rapidly build up molecular‐scale complexity with minimum effort.^[^ ^55^ ^]^ A gel has genuine potential to mediate multiple reactions, and this topic will be returned to in later sections of the review.

In summary, therefore, self‐assembly is an effective way of modifying the performance of catalytic motifs and creating nanomaterials that can outperform isolated small molecules. Specifically, LMWGs can exhibit enhanced or modified catalytic performance through a variety of mechanisms:

- Self‐assembled dimer/aggregate is itself the active catalyst, hence self‐assembly is a prerequisite for effective catalysis.
- Self‐assembly changes the pKa of active groups by placing them in unique environments, assisting catalysis.
- Self‐assembly can modify active site accessibility leading to steric control over catalysis and changing reaction selectivity.
- Self‐assembled hydrophobic domains help solubilize/organize hydrophobic reagents close to the active site, enhancing catalysis.

### 2.2 Synergistic Effects in Multicomponent Gels

**Figure 5:**
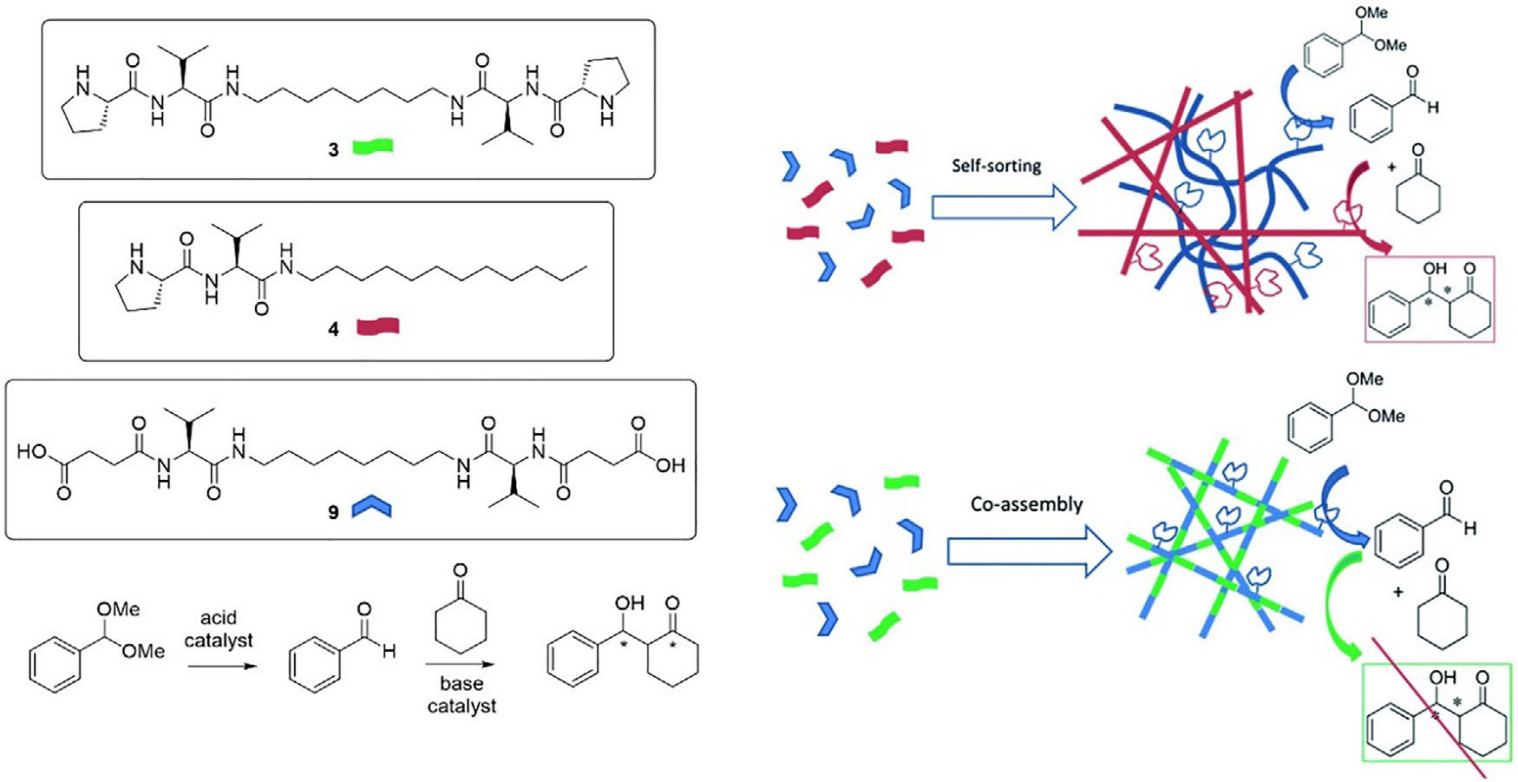
Succinic acid–modified gelator **9** can catalyze acid‐mediated acetal deprotection, whereas proline‐based gelators **3** and **4** can catalyze the aldol reaction. Combining **4** and **9** in a multicomponent gel with self‐sorting allows both catalytic sites to retain their reactivity, enabling a two‐step reaction to occur (top right). Combining **3** and **9** in a multicomponent gel with a coassembly mechanism loses activity and the two‐step reaction does not take place (bottom right). Figure adapted from Reference [56] with permission of the Royal Society of Chemistry.

Going beyond their work with tandem reactions in gels, Escuder and coworkers realized that it may be possible to assemble gels that would combine multiple components with orthogonal, mutually destructive reactivities in a single integrated material.^[^ ^56^ ^]^ This concept emerged from extensive work from the wider supramolecular gels community on multicomponent gels, which had led to a detailed understanding of the ways in which they can assemble.^[^ ^57^, ^58^, ^59^ ^]^ Two different LMWGs can, if appropriately designed, each assemble into their own separate nanostructure—a process referred to as self‐sorting. In this way, Escuder and coworkers combined a succinic acid–functionalized LMWG (**9**) with basic proline‐based LMWG **4** (Figure 5). Normally, such molecules in solution would simply neutralize one another, preventing both from being active in the same system. However, once restrained within self‐assembled nanofibers, these units coexisted. Escuder and coworkers demonstrated that the self‐sorted two‐component gels catalyzed a two‐step reaction process, with self‐assembled **9** catalyzing an acid‐mediated acetal deprotection and self‐assembled **4** catalyzing a subsequent base‐mediated aldol reaction (Figure 5). Fascinatingly, when related LMWGs (**9** and **3**) were coassembled into a combined nanostructure, rather than being self‐sorted into independent networks, they were no longer proficient in catalyzing the two‐step process. This demonstrates that nanoscale separation of reactive groups was a key part of catalytic function. This elegant example not only demonstrates how mutually reactive species can be harnessed in a single integrated nanomaterial but also illustrates how such gels can be useful reaction vessels for performing multistep reaction processes in one‐pot. This concept of using gels for “reaction engineering” is one we return to in Sections 5 and 6.

In another exploration of sorting effects on catalysis, Escuder and coworkers explored the three‐component Mannich reaction between benzaldehyde, aminobenzene, and cyclohexanone, once again simultaneously catalyzed by acidic and basic LMWGs.^[^ ^60^ ^]^ In this case, however, when the two LMWGs self‐sorted, they were not proficient, indeed they competed with one another for the Mannich reaction. However, in the presence of soluble molecules of succinic acid–based **9**, the mobility of the acid allowed it to cooperate with the solid‐like self‐assembled fibers of **4** and hence provide much improved selectivity (*anti/syn* 95:5). This demonstrates that the organization of individual components within a multicomponent gel is not always a simple issue when developing systems for applications in catalysis, with the optimal solution depending on both self‐assembled nanostructures and the requirements of the reaction transition state(s).

Learning from the way in which enzymes organize amino acids to achieve functional catalysis, Qi and coworkers used a coassembly method to endow self‐assembling multicomponent gels with greater functionality.^[^ ^61^ ^]^ They modified Fmoc‐FF, a known highly effective small peptide hydrogelator based on phenylalanine protected with the 9‐fluorenylmethyloxycarbonyl (Fmoc) group, with additional amino acids to create Fmoc‐FFS, Fmoc‐FFH, and Fmoc‐FFD. The choice of Ser/His/Asp (S/H/D) as the additional amino acids reflects the fact that this is the catalytic triad found in many natural hydrolase enzymes. The researchers found that Fmoc‐FFH was a much more effective catalyst than free histidine for the hydrolysis of 4‐nitrophenyl acetate. By coassembling the three different peptides at a 1:1:1 ratio into a gel, the researchers could incorporate all three amino acids onto a nanostructured support, however, disappointingly catalytic activity was reduced compared with Fmoc‐FFH alone. Careful optimization of the ratio of the different components led to the discovery that a 40:1:1 (His/Ser/Asp) system was 1.7 times more active than Fmoc‐FFH alone, suggesting that the different amino acids may, to some extent, collaborate with the histidine in this coassembled system. Impressively, the authors then demonstrated that “imprinting” the gel by forming it in the presence of the substrate (4‐nitrophenyl acetate) further improved the activity by 7.8 times, suggesting imprinting can preorganize the different amino acids required for optimal catalysis. Taking a similar multicomponent strategy, Roy and coworkers explored hydrogels based on combining Nap‐F, with the basic amino acids H, K, and R in different ratios.^[^ ^62^ ^]^ They found that the presence of lysine induced helical fibril morphology and enhanced catalytic esterase‐like activity.

In addition to combining multiple LMWGs, it is also possible to combine catalytic LMWGs with other catalytically active agents to achieve enhanced reactivity. For example, Ranganath and coworkers combined a chiral self‐assembling LMWG with NiO nanoparticles, to create a hybrid material that could catalyze the asymmetric Michael addition of malonates with chalcones in high yields and with excellent enantioselectivity.^[^ ^63^ ^]^ Although bare NiO nanoparticles could catalyze this reaction on their own, the enantioselectivity was improved for the gel‐modified particles as a result of the LMWG chirality, with the precise outcome depending on the structure of the substrate.

### 2.3 Prebiotically Relevant Catalysis Mediated by LMWGs

**Figure 6:**
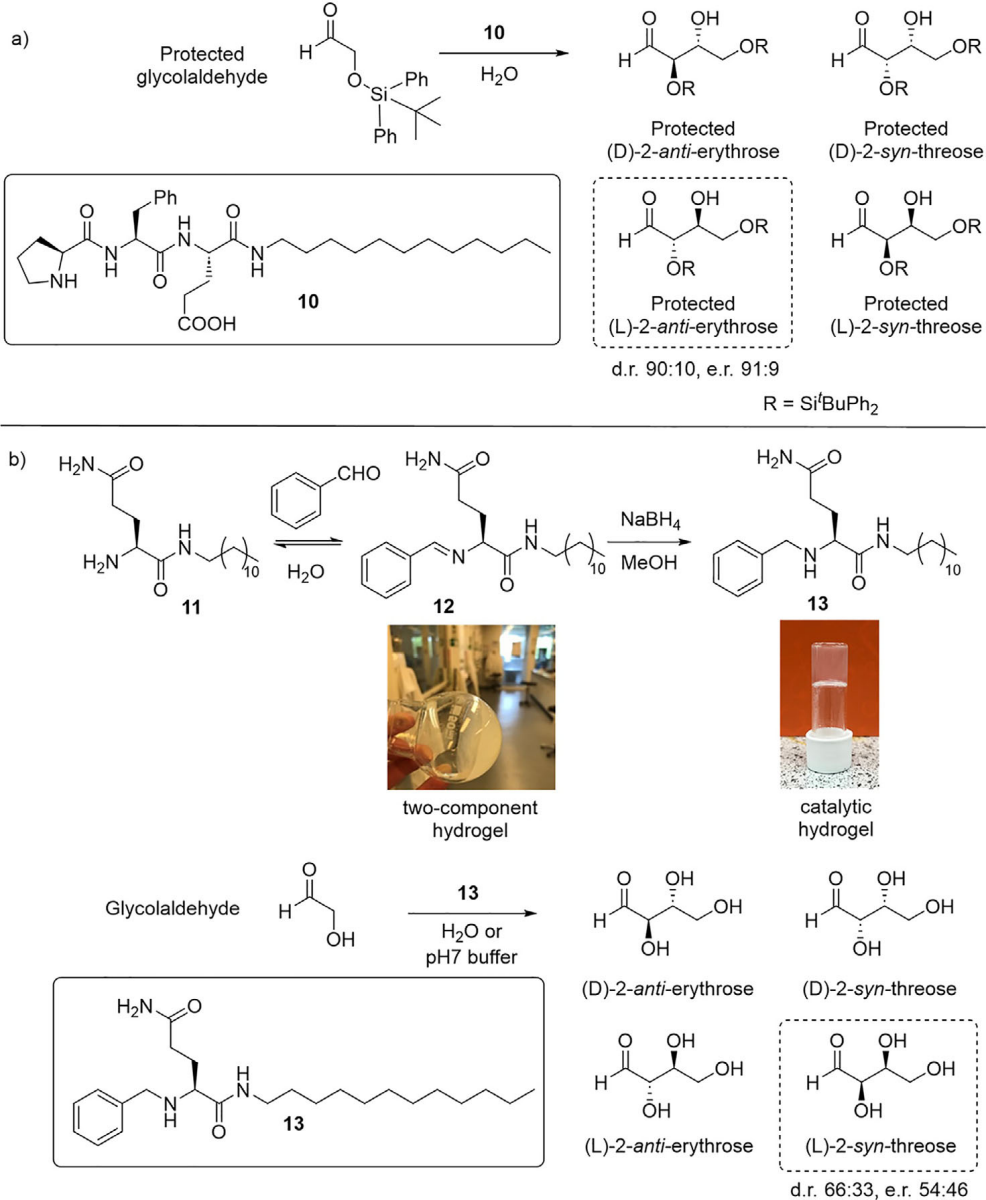
a) Proline‐based LMWG **10** catalyzes the aldol condensation of protected glycolaldehyde in water to yield protected erythrose/threose with excellent stereoselectivity, giving protected l‐2‐*anti*‐erythrose as the major product. b) Condensation between modified glutamine **11** and benzaldehyde followed by reduction of the Schiff base product **12** provides LMWG **13**, which catalyzes the aldol condensation of unprotected glycolaldehyde in water, giving l‐2‐*syn*‐threose as the major product with a degree of stereoselectivity. Figure adapted from Reference [70] with permission of the American Chemical Society.

There is considerable general interest in using organocatalysts in prebiotically‐relevant processes, with the suggestion being that simple low‐molecular‐weight systems may have played an important role in catalyzing reactions that led to the molecular building blocks required for the emergence of life on the early Earth.^[^ ^64^, ^65^ ^]^ As described above, simple LMWGs can catalyze aldol bond‐forming reactions, and it is notable that such reactions are often argued to have played a key role in the development of molecular complexity on the early Earth.^[^ ^66^, ^67^, ^68^ ^]^ With this in mind, in 2016, Escuder and coworkers tested proline‐based LMWG **10** as the catalyst for a prebiotically relevant aldol reaction—the self‐condensation of glycolaldehyde (Figure 6a).^[^ ^69^ ^]^ This reaction gives rise to a mixture of erythrose and threose, sugars required in the synthesis of important biomolecules. In unprotected form, glycolaldehyde would not react with the hydrogel formed by **10**. Conversely, with a TIPS or TDBS protecting group on the alcohol, this reaction proceeded in excellent yield and selectivity. The major product of the reaction was protected l‐2‐*anti*‐erythrose, which was obtained with remarkable stereoselectivity (d.r. 90:10, enantiomeric ratio (e.r.) 91:9). The authors suggested that the hydrophobicity of the protected substrate helped it accumulate near the reactive site via the mechanism described in Section 2.1.

With a similar ultimate goal in mind, my team worked with that of Clarke also here in York to develop hydrogels capable of performing an aqueous aldol self‐condensation reaction with glycolaldehyde, targeting unprotected, water‐soluble “prebiotic” glycolaldehyde as our reaction substrate (Figure 6b).^[^ ^70^ ^]^ Initially, we had developed a self‐assembling glutamine derivative (**11**) that formed a weak gel in the hope it would act as an aldol catalyst in reactions between cyclohexanone and 4‐nitrobenzaldehyde. The reaction was promoted, but the LMWG also reacted directly with the aldehyde to form an imine (**12**), which subsequently assembled into a much more effective sample‐spanning gel. On reduction of this imine, we obtained a highly effective hydrogelator **13** (minimum gelation concentration 0.03% wt vol^−1^) with a secondary amine suitable for enamine‐type catalysis. Pleasingly, the hydrogel formed by **13** promoted the conversion of unprotected glycolaldehyde to erythrose/threose both in pure water and in pH 7 buffer, with excellent conversion (≈70%). The major product of the reaction was determined to be l‐2‐*syn*‐threose, with a degree of stereoselectivity (diastereomeric ration [d.r.] 66:33, enantiomeric ratio [e.r.] 54:46). Notably, this is a different stereoisomeric product to that observed (in protected form) by Escuder and coworkers,^[^ ^69^ ^]^ indicating that the different catalytic units have different preferences. However, it is also possible that the bulky protecting group in Escuder's work may influence selectivities. Importantly, in its nonassembled form, LMWG **13** was very inefficient in this reaction (only ≈5% conversion)—gel assembly, therefore, significantly enhances catalysis. Given the high water‐solubility of glycolaldehyde, it is not possible that hydrophobicity enhances reactivity, and it is suggested that the highly organized nanostructures formed by the LMWG, place the catalytic sites close together, hence enhancing reactivity. Clearly, this work demonstrates that self‐assembled structures can be proficient in catalysis of simple reactions that may have been important on the early Earth, in prebiotically relevant conditions.

### 2.4 Toward Synthetic Cells/Protocells with LMWGs

The gel‐promoted catalysis of a prebiotically important process described in Section 2.3 above hints at the possibility that on the early Earth, the self‐assembly of organocatalysts into nanostructured materials may have helped drive reaction processes and amplify some of the key components required for life. Furthermore, it has been proposed that gels may have constituted some of the earliest cells, providing a degree of separation between “interior” and “exterior” even in the absence of a membrane, allowing important or reactive components to accumulate within them.^[^ ^71^, ^72^, ^73^, ^74^, ^75^ ^]^ It has even been suggested that prebiotic gel droplets may have carried key building blocks of life between planets as part of the panspermia hypothesis and then played a functional role in establishing life in the new environment.^[^ ^76^ ^]^ Notably, the cytoplasm of modern cells is a gel‐like medium, which helps mediate the intracellular trafficking of molecules, as well as controlling the location of interior cellular substructures.^[^ ^77^, ^78^ ^]^ There is, therefore, considerable emerging interest in the ability of gels to mimic a range of cellular processes, including reactivity profiles.^[^ ^79^ ^]^

**Figure 7:**
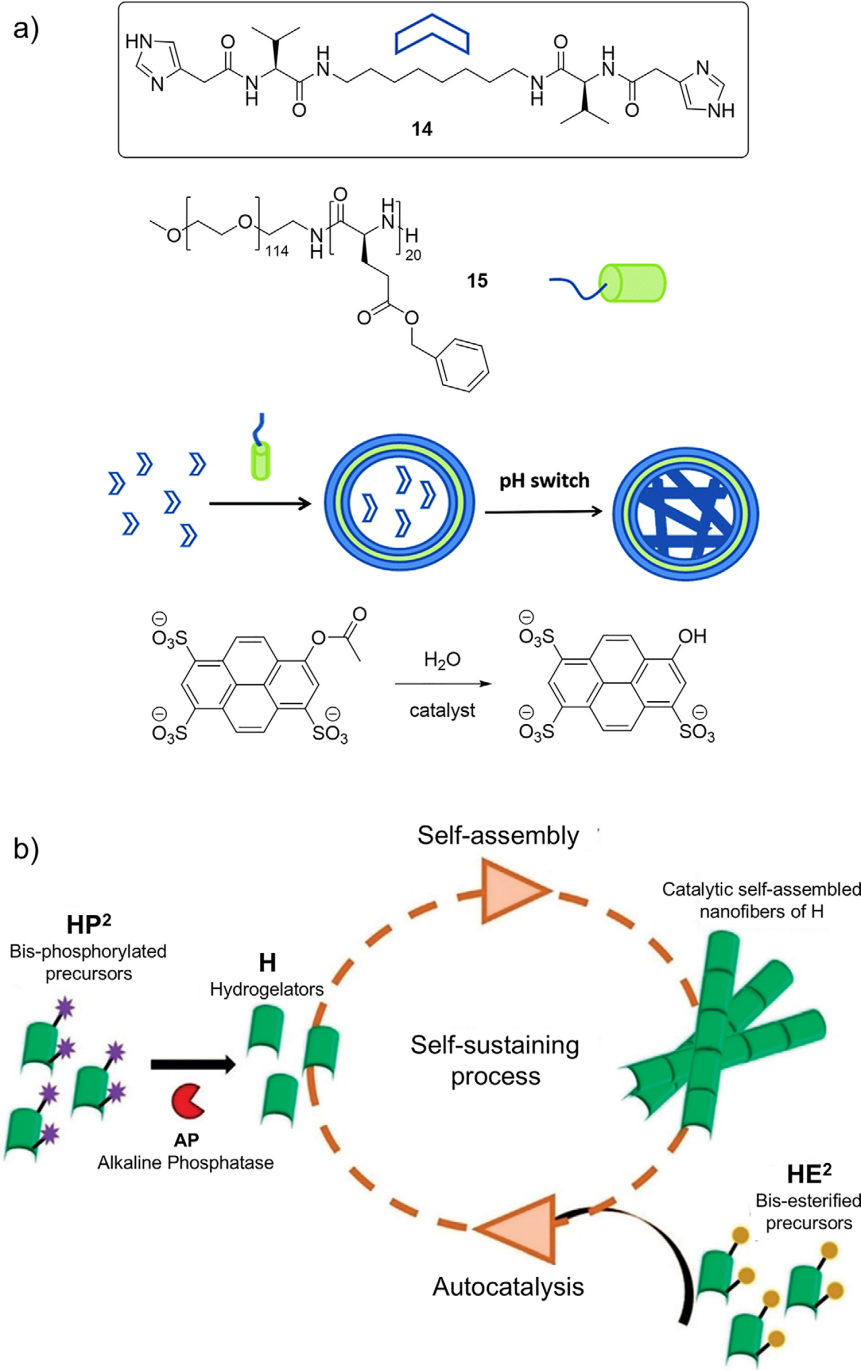
a) Imidazole‐functionalized LMWG **14** assembles via a pH switching mechanism inside a polymerosome formed by compound **15**—the self‐assembled gel fibers within the polymerosome are active catalysts for the ester hydrolysis of a soluble pyrene derivative (bottom). Figure adapted from Reference [80] with permission from the Royal Society of Chemistry. b) Hydrogelator H, produced by trace amounts of AP hydrolyzing bis‐phosphorylated precursor HP^2^, self assembles into nanofibers that subsequently catalyze the hydrolysis of bis‐esterified precursor HE^2^, establishing an autocatalytic self‐assembly cycle. Figure reproduced from Reference [82] with permission of Wiley‐VCH.

With a close eye on cellular mimicry, Escuder and coworkers assembled pH‐responsive LMWG **14** incorporating an imidazole unit within a polymerosome assembled from block copolymer **15**, based on poly(ethyleneglycol) (PEG) as the hydrophilic block and poly(γ‐benzyl‐l‐glutamate) (PBG) as the hydrophobic block (Figure 7a).^[^ ^80^ ^]^ This approach, i.e., assembling an LMWG within a liposome, builds on foundational work from van Esch and coworkers on the orthogonal assembly of LMWGs and surfactants.^[^ ^81^ ^]^ To fabricate the system, Escuder and coworkers incorporated the LMWG within the polymerosome in soluble form in acidic conditions and then raised the internal pH until self‐assembly occurred. They coencapsulated an ester derivative of a soluble pyrene and showed that as the gel fibers assembled within the polymerosome, the ester was hydrolyzed. Fascinatingly, the polymersome confines this activity to a micro/nanometric length scale, and although this is, of course, not a protocell, it does begin to illustrate how orthogonal self‐assembly can yield systems with spatially controlled reactivity profiles, which lead to chemical composition differences across a membrane interface, a key aspect of protocell‐type behaviour.

Autocatalysis may have also played a key role in the evolution of life on the early Earth. In an autocatalytic supramolecular gel, the assembly of gel fibers catalyzes the formation of the component required to assemble the gel. In this way, a small amount of a self‐assembling LMWG effectively amplifies its own production. This was clearly illustrated by the work of Fores and coworkers (Figure 7b),^[^ ^82^ ^]^ who reported that a domino sequence of events starts from the generation of a self‐assembling peptide (Fmoc‐GFFYGHY, H) from a phosphorylated precursor (HP^2^) by trace amounts of a hydrolytic enzyme (AP, 10^−18^ m). The researchers had previously shown that these self‐assembled nanofibres could catalyze the hydrolysis of nonactivated esters^[^ ^83^ ^]^—a relatively unusual and powerful feature, given that most hydrolytic gels have been applied to highly‐activated systems (e.g., those with 4‐nitrophenyl groups). Therefore, when exposed to an esterified peptide precursor (HE^2^), the self‐assembled fibers catalyzed its hydrolysis and in this way, more peptide was generated, which in turn assembled into additional catalytic self‐assembled nanofibers, with the overall system becoming autocatalytic. This type of process may have been relevant in the amplification of building blocks on the early Earth. It was also found that the growing fibers had a preferential growth orientation perpendicular to the initiating surface, with a gradient of fiber density from the surface to the top. This orientational fiber growth offers an interesting analogy with the anisotropic properties of biological tissues. Indeed, the spatial resolution and patterning of gel‐phase materials is an important current topic,^[^ ^84^ ^]^ explored in more depth later in this review.

It seems likely that research into gels as protocells will continue apace, in part because such systems have fundamental significance in terms of understanding the origins of life, but also because microscale encapsulated reactive gels may have a wide range of technological applications in a variety of industrial settings.

## 3 Supramolecular Gels in Enzyme Catalysis

Moving away from the concept that the gel network itself is catalytically active, it is also possible to encapsulate catalytically active units within a self‐assembled gel to enhance reaction outcomes. There are various potential benefits this can offer, such as improving the useability of the catalyst, introducing recyclability to the system, or modifying/enhancing reactivity. Once again, the latter is the primary focus here of the examples selected here. As relatively large molecules, enzymes are interesting active species to encapsulate because their size essentially prevents them from diffusing through or out of the gel.^[^ ^85^ ^]^ This means enzymes trapped in a supramolecular gel network can essentially be considered immobilized on the molecular scale. This is in sharp contrast to soluble, small molecule reagents and products, which can rapidly diffuse through the gel matrix, allowing gels to offer a degree of spatial control over enzyme‐mediated reactions.

### 3.1 Enhancing Enzyme Stability and Activity Using LMWGs

**Figure 8:**
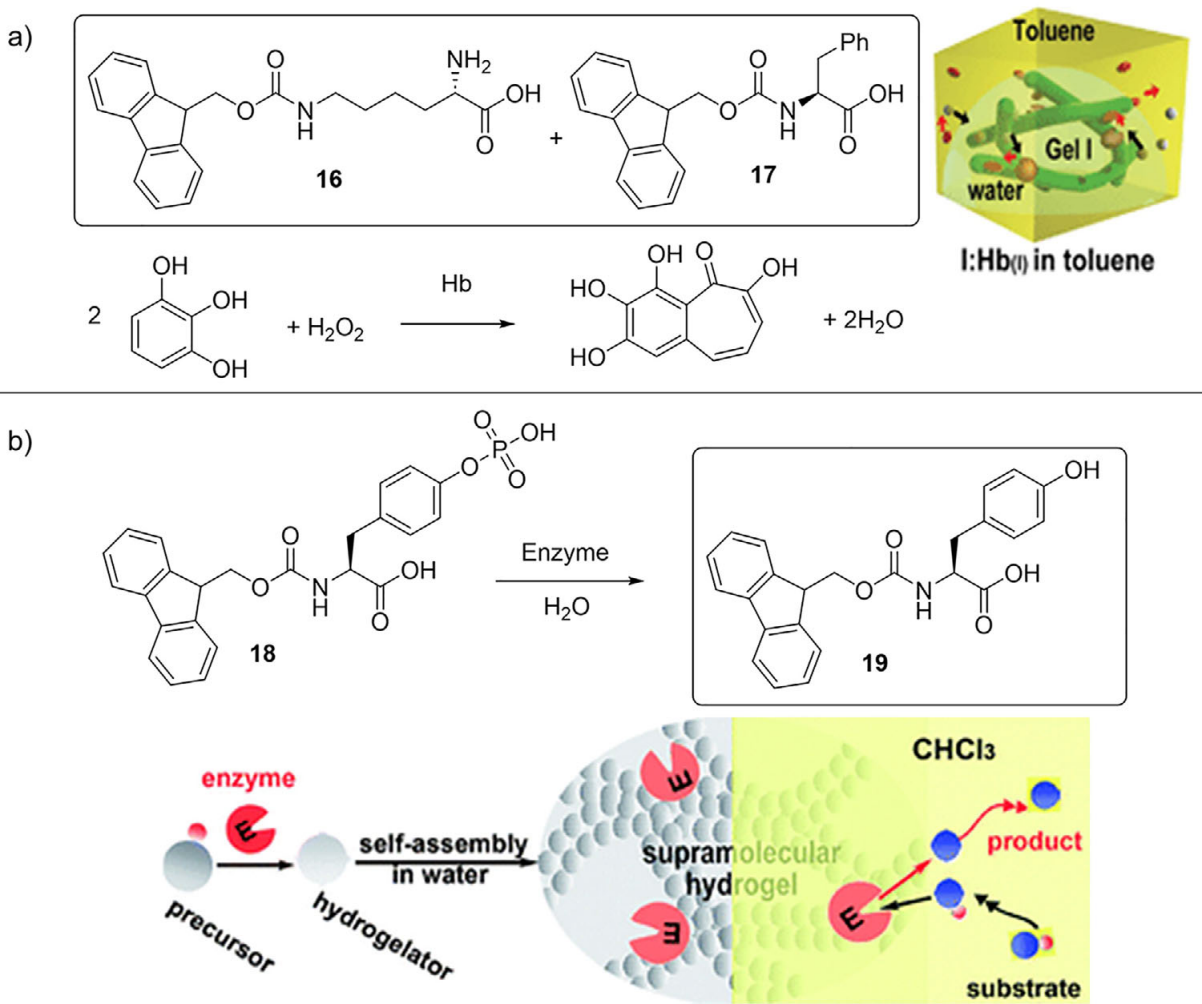
a) Two‐component hydrogel based on peptides **16** and **17** was assembled in the presence of hemoglobin and used for the oxidation of pyrogallol to purpurogallin in toluene as bulk solvent, with the enzyme exhibiting “superactivity”. Scheme adapted from Reference [86] with permission from the Royal Society of Chemistry. b) Phosphorylated precursor **18** was converted to LMWG **19** by an acid phosphatase enzyme, which became encapsulated within the gel. The enzyme‐loaded hydrogel was subsequently used for hydrolysis of 4‐nitrophenyl phosphate (shown in schematic form as large blue circle with a small red circle connected) in organic solvents. Scheme adapted from Reference [87] with permission from the Royal Society of Chemistry.

In 2007, pioneering work from Xu and coworkers carefully combined enzymes with a warm two‐component LMWG solution (**16**+**17**), and on cooling to room temperature, a peptide hydrogel with encapsulated enzymes was assembled.^[^ ^86^ ^]^ As one example, they encapsulated hemoglobin within the gel and used it to catalyze the H_2_O_2_‐mediated oxidation of pyrogallol to purpurogallin in toluene (Figure 8a). The enzyme‐encapsulated gel was 8 times more active than hemoglobin in water, an effect they described as “superactivity”. Interestingly, the improvement was significantly greater than that observed in a standard cross‐linked polymer hydrogel. They suggested that several factors were responsible: i) hydrophilicity promotes the transfer of the pyrogallol substrate across the toluene/water interface into the supramolecular hydrogel, (ii) the amphiphilic nature of assembled gel fibers may help substrates approach (and products leave) the encapsulated enzyme, and (iii) the relatively large pore size in the supramolecular gel facilitates mass transport. The stability of the enzyme was also significantly improved in the hydrogel, compared with bulk water. The ability to use enzymes effectively in organic solvents is of value because many organic substrates have poor solubility in the enzyme's preferred aqueous media. As such, encapsulating enzymes within a hydrogel, and subsequently using them in organic media, offers a powerful approach to broadening the scope of enzyme‐mediated reactions.

Rather than using a heat–cool cycle to generate the gel, which risks denaturing the enzyme, it is also possible to trigger gel assembly in the presence of the enzyme.^[^ ^87^ ^]^ Xu and coworkers used acid phosphatase to convert precursor **18** into LMWG **19**, hence assembling the gel in situ, while simultaneously encapsulating the acid phosphatase enzyme (Figure 8b). The ability of the encapsulated enzyme to then catalyze the hydrolysis of 4‐nitrophenylphosphate to 4‐nitrophenol was tested. Once again, the activity of the hydrogel in an organic phase was significantly enhanced compared to the free enzyme—in chloroform, ≈100 times. These high activities were ascribed to the cooperative effect of the amphiphilic nanofibers in the hydrogel and phase transfer between the organic solvent and the water in the hydrogel.

Das and coworkers explored cytochrome c in self‐assembling amphiphilic LMWG systems, showing up to 350‐fold enhancement in rate in the H_2_O_2_‐mediated oxidation of pyrogallol.^[^ ^88^ ^]^ They also went on to fabricate microgel versions of this encapsulated enzyme (see Section 6). Marr and coworkes encapsulated lipase enzymes in supramolecular ionic liquid gels and demonstrated they were active in the hydrolysis of 4‐nitrophenyl butyrate.^[^ ^89^ ^]^ As such, the application of enzymes in gels is not limited to hydrogels, which may expand the potential use in organic synthesis across a wider variety of substrates.

**Figure 9:**
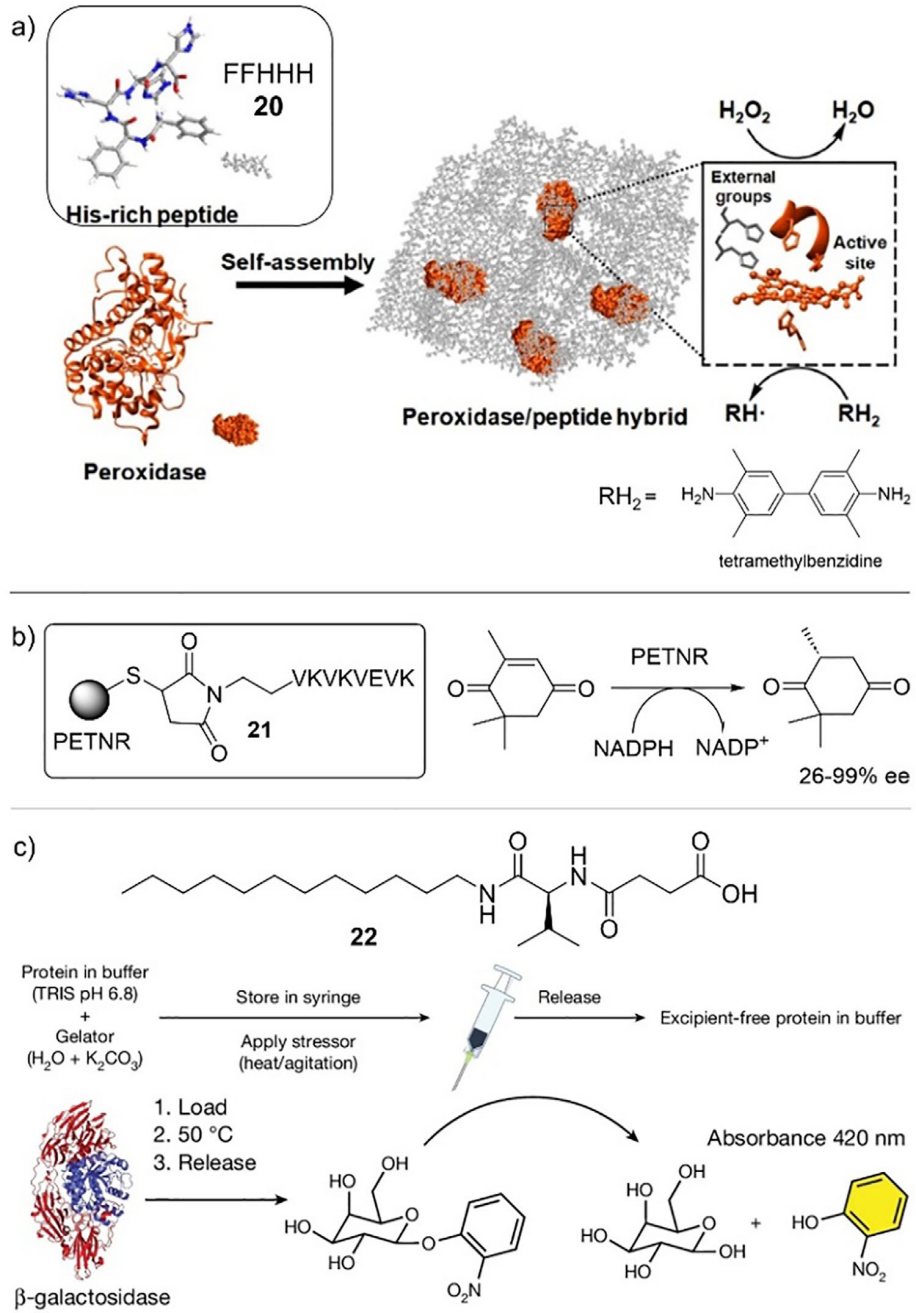
a) Histidine‐rich peptide LMWG **20** is coassembled with a heme‐dependent peroxidase enzyme to create a gel that is active in the H_2_O_2_‐mediated oxidation of tetramethylbenzidine (RH_2_). The histidine groups on the LMWG approach the heme in the active site to enhance reactivity. Figure reproduced from Reference [90] with permission from Springer. b) Compound **21** conjugates pentaerythritol tetranitrate reductase (PETNR) to a self‐assembling peptide and the resulting gel reduces ketoisophorone to (*R*)‐levodione using nicotinamide adenine dinucleotide phosphate (NADPH) as a cofactor. c) LMWG **22** assembles into gels in the presence of proteins/enzymes stabilizing the enzyme against thermal stress or agitation and then releases them via syringe injection. Within the gel, β‐galactosidase retains its activity for the hydrolysis of 2‐nitrophenyl‐galactoside, even after heating at 50 °C. Figure adapted from Reference [91] with permission from Springer Nature.

It is also possible that direct, specific interactions between a self‐assembled LMWG network and an encapsulated enzyme may influence reaction outcomes. To demonstrate this, Wang and coworkers combined a self‐assembling histidine‐rich LMWG (**20**) with a heme‐dependent peroxidase enzyme (Figure 9a).^[^ ^90^ ^]^ The results indicated that the self‐assembling peptide altered the enzyme conformation, promoting transitions between the resting and the intermediate states of the heme. This increased the turnover rate of the enzyme for the H_2_O_2_‐mediated oxidation of tetramethylbenzidine up to 3‐fold. It was proposed that the histidine residues from the self‐assembling peptide approached the active‐site heme group, accelerating the catalytic cycle. In the future, computational approaches may allow the precise design of self‐assembling LMWGs engineered to interact synergistically with encapsulated enzymes and direct reactivity in smart ways.

To further stabilize enzymes within the gel and prevent leaching, Miller and coworkers created self‐assembling peptides in which the enzyme was directly conjugated to the peptide (Figure 9b).^[^ ^92^ ^]^ The enzyme pentaerythritol tetranitrate reductase was attached to a VKVKVEVK self‐assembling peptide either via cysteine–maleimide type conjugation or through a genetic expression approach. In both cases, the enzyme was much less prone to leaching but still showed effective conversion of ketoisophorone to (*R*)‐levodione. Furthermore, the enzyme was significantly more robust both thermally and temporally in the gel

Building on the concept that gels can stabilize and protect biomolecules from denaturing, recent high‐profile research suggested the value of this approach with medicinally active proteins.^[^ ^91^ ^]^ Such systems often suffer from thermal instability, meaning cold chains must be ensured for transport and storage, which can be challenging in some parts of the world. Adams, Gibson and coworkers designed a stiff hydrogel based on LMWG **22** that stabilizes proteins against thermal denaturation even at 50 °C and delivers pure, excipient‐free protein by mechanical release from a syringe (Figure 9c). To demonstrate the stabilization principle, they encapsulated β‐galactosidase and found that after storage under thermal stress at 50 °C for 7 days, it retained 97% of its catalytic function in the hydrolysis of 2‐nitrophenyl‐galactoside. They also demonstrated that gel‐encapsulated insulin could be stored at 25 °C, rather than the usual low‐temperature conditions. They even posted their gels through the UK postal service to show they survived handling. This work starts to show how control over biomolecular activity in gels may move beyond synthetic chemistry into therapeutic applications.

In summary, incorporating enzymes in gels can modify their stability and activity. This can occur on steric grounds as a result of the gel network limiting enzyme diffusion and/or preventing protein unfolding/denaturing. Furthermore, placing the enzyme in a hydrogel or ionogel helps maintain its stability by providing an appropriate local environment, yet the gel allows the enzyme to then be used in an organic solvent where it may not normally be viable. The gel network can also play a more explicit role as a result of direct interactions between LMWG and encapsulated enzyme. Exciting recent work clearly indicates that although these concepts have been established for some time, enzyme‐loaded supramolecular gels have considerable future potential.

### 3.2 Creating Artificial Enzymes Using LMWGs

**Figure 10:**
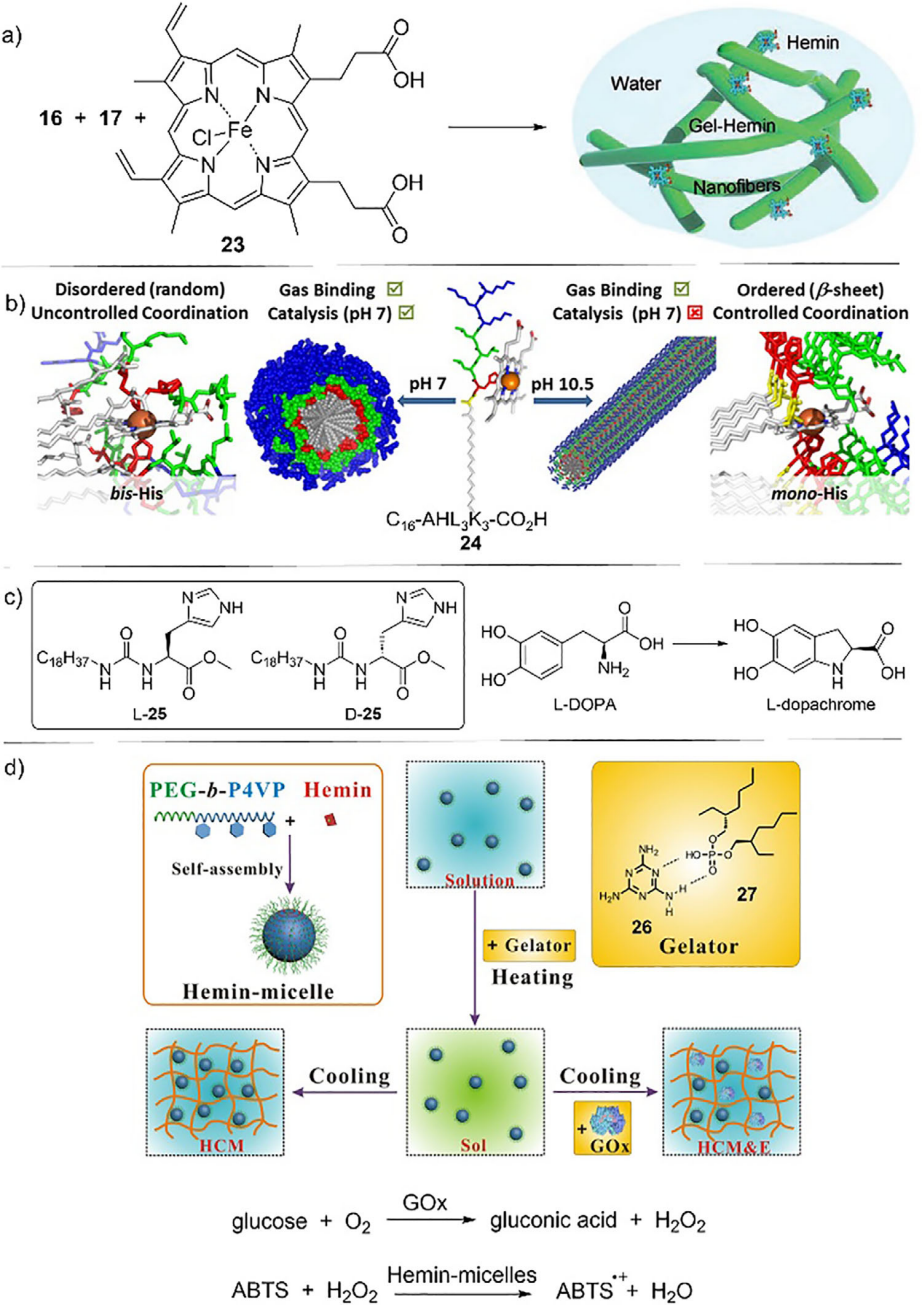
a) Two‐component gel based on **16** and **17** (see Figure 8a for structures) incorporates hemin **23** to create a hybrid gel capable of HRP‐type oxidation. Figure adapted from Reference [91] with permission from Wiley‐VCH. b) Self‐assembling peptide **24** can assemble with hemin into micelles with bis‐histidine hemin ligation at pH 7 (left) or gel nanofibers with mono‐histidine hemin ligation at pH 10.5 (right). The micelles are more proficient peroxidase‐type catalysts. Figure reproduced from Reference [95] with permission from the American Chemical Society. c) Enantiomeric LMWGs l‐**25** and d‐**25** were coassembled with hemin and the resulting gels catalyzed l‐DOPA oxidation. d) Block copolymer PEG‐*b*‐P4VP was assembled into micelles with hemin and mixed with **26** and **27**, which form a two‐component supramolecular gel. This gel could also be loaded with glucose oxidase (GOx). This multicomponent system catalyzes the oxidation of glucose and subsequent oxidation of ABTS. Figure reproduced from Reference [96] with permission from the American Chemical Society.

In addition to immobilizing enzymes in gels, it is possible to use active subunits, which once encapsulated in a gel, behave like an artificial enzyme, with the structure of the LMWG being somewhat like an enzyme superstructure in terms of stabilization and organization. In early work, Xu and coworkers immobilized hemin (**23**) within their two‐component peptide hydrogel based on **16** and **17** (Figure 10a) to create a system that could mimic horseradish peroxidase (HRP).^[^ ^93^ ^]^ The gel reached about 60% of the activity of HRP for oxidation of pyrogallol to purpurogallin, exceeding the performance of hemin in water by a factor of 17.6. Such systems can be considered as artificially constructed enzymes. Further optimization allowed these researchers to create a synthetic system that exhibited 90% of the activity of HRP for an oxidation reaction in toluene.^[^ ^94^ ^]^ This was achieved by using a different heme with modified distal substituents.

Fry and co‐workers also encapsulated hemin in supramolecular materials (Figure 10b).^[^ ^95^ ^]^ In their case, their self‐assembling system (**24**) contained a histidine group. Histidine is a potential axial ligand for the iron center in hemin. They found that if the peptide assembled into micelles, bis‐histidine ligation occurred, but if it assembled into gel fibers, mono‐histidine coordination was the result. These morphological changes altered the peroxidase‐type activity of the hemin. In this case, the micelles actually had higher activity than the gel, but nonetheless, this demonstrates how direct interactions between a self‐assembled system and an encapsulated catalytic unit can modify activity, indicating how careful LMWG design or multicomponent approaches can help direct specific reaction outcomes.

Liu, Zhang and coworkers went on to combine single enantiomer histidine amphiphile LMWGs (**25**) with hemin to create gels and tested their ability to catalyze the oxidation of the chiral substrate l‐DOPA (Figure 10c).^[^ ^97^ ^]^ Systems based on opposite enantiomers of the histidine amphiphile catalyzed the reaction to different extents, with d‐**25**‐hemin having *k*_cat_ values ≈times greater than l‐**25**‐hemin. It was argued that the chiral amphiphile provides a chiral environment for the hemin and the d‐**25**‐hemin system shows preferential enantioselective binding and hence oxidative catalysis of l‐DOPA. Hemin has also been incorporated into other supramolecular gels, for example, those based on G‐quartets. These gels were demonstrated to exhibit peroxidase type activity in the oxidation of tetramethylbenzidine.^[^ ^98^ ^]^

In interesting work, Shi and coworkers created a multifunctional system capable of catalyzing cascade reactions (Figure 10d).^[^ ^96^ ^]^ Specifically, they stabilized hemin in a block copolymer micelle and then combined these micelles with a supramolecular two‐component gel (melamine **26** + di(2‐ethylhexyl) phosphoric acid **27**) that was also used to stabilize glucose oxidase (GOx). This multicomponent system enabled glucose to be oxidized by oxygen, producing the toxic intermediate H_2_O_2_, which was then eliminated as hemin used it to catalyze oxidation of 2,2′‐azino‐bis(3‐ethylbenzothiazoline‐6‐sulfonate) (ABTS), producing a dark green radical cation. A solution‐phase mixture of the individual components was incapable of achieving this process. Like the examples in Section 2.2, this demonstrates how supramolecular gels offer a powerful way of stabilizing orthogonal reactive systems to promote multistep reaction processes. In later work, glucose oxidase and hemin were both incorporated within a self‐assembling peptide gel, and it was again demonstrated they had enhanced activity for cascade reactions.^[^ ^99^ ^]^ Specifically, glucose was oxidized by GOx to produce H_2_O_2_, which was in this case used by hemin to convert pyrogallol into purpurogallin. Furthermore, the peptide LMWG was an active controlling factor as it incorporated an azo‐benzene group, with photo‐induced E→Z switching converting the gel into a sol, decreasing activity, which then partly recovered if the azobenzene was switched back from Z→E, reassembling the gel. The system could be cycled a few times, demonstrating how external stimuli can influence reaction outcomes, a theme explored further in Section 6.

There is considerable general interest in the ability of gels to produce and deliver reactive oxygen species (ROS), with such systems having potential uses in synthetic chemistry and emergent biomedical applications.^[^ ^100^, ^101^ ^]^ A biomimetic approach is often taken, for example, Perez‐Garcia, Amabilino and coworkers incorporated 5,10,15,20‐tetrakis(4‐carboxylatephenyl)porphyrin (TCPP) within a supramolecular gel formed by a bis‐imidazolium amphiphilic salt.^[^ ^102^ ^]^ The singlet oxygen generation was indicated via oxidation of 9,10‐anthracenedyl‐bis(methylene)dimalonic acid, which can be oxidized by singlet oxygen. On irradiation, the oxidation of this probe was ≈14 times faster in the gel than in solution. TCPP interacts electrostatically with the self‐assembled gel nanofibers, and combined with it being held within a gel network, this prevents the aggregation of TCPP, hence enhancing the production of ROS.

**Figure 11:**
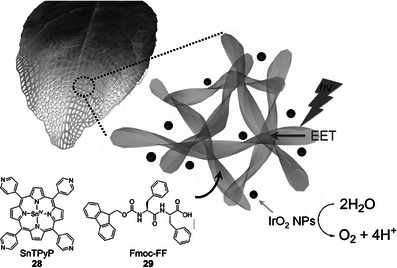
Peptide LMWG Fmoc‐FF (**29**) was combined with tin porphyrin SnTPyP **28**. The resulting coassembly brought the porphyrins into close proximity, and when combined with IrO_2_ nanoparticles, enabled excitation energy transfer (EET) that could drive the photoreduction of water. Figure reproduced from Reference [103] with permission from Wiley‐VCH.

In addition to studies of oxidative chemistry with hemin‐loaded or porphyrin‐loaded gels, hydrogels have also been explored for their potential to catalyze processes important in energy technology, such as photocatalytic water oxidation (Figure 11).^[^ ^103^ ^]^ Water‐splitting is challenging because it requires multiple‐electron transfer coupled with proton transfer at a minimum potential of 0.81 V versus normal hydrogen electrode (NHE) at pH 7. Natural photosystems overcome this by spatially arranging active groups in catalytic clusters. On combining Fmoc‐FF self‐assembling LMWG (**29**) with metalloporphyrin **28**, the porphyrins were incorporated into the self‐assembled gel fibers in close enough proximity that excited energy transfer between them was enabled, i.e., gel assembly enabled the formation of “catalytic clusters”. When further combined with IrO_2_ nanoparticles, photocatalytic water oxidation was enhanced ≈3.7 times compared to a physical mixture of the individual components. Increasing the loading of metalloporphyrin **28** enhanced the process as the porphyrins came into even closer proximity in the hydrogel.

In recent research, a synthetic gel system was created that could mimic (and improve) the performance of lignin degradation enzymes.^[^ ^104^ ^]^ Lignin is a major component of plant cell walls and its effective processing is important for biomass conversion. A self‐assembling peptide gel in an ionic liquid that binds Cu(II) ions was developed. Lignin solubility is enhanced in the ionic liquid medium, and the copper center leads to oxidase‐mimetic lignin degradation. Interestingly, this wholly synthetic system was catalytically proficient up to 75 °C, which is significantly better than typical enzymes, demonstrating the potential power of the supramolecular approach.

This general approach to supramolecular “nanozymes”,^[^ ^105^ ^]^ in which self‐assembly provides active units with stability and introduces the potential to modulate them via noncovalent interactions, is a strategically powerful way of creating complex synthetically active systems from simple, commercially viable building blocks. In addition to enhancing the performance of known catalysts, it is anticipated that wholly new types of catalysis may be achieved using minimal active building blocks in gels, providing innovative tools for next generation synthetic chemistry.

## 4 Supramolecular Gels to Stabilize Reactive Species

### 4.1 Stabilizing Reactive Catalytic Nanoparticles in Gels

**Figure 12:**
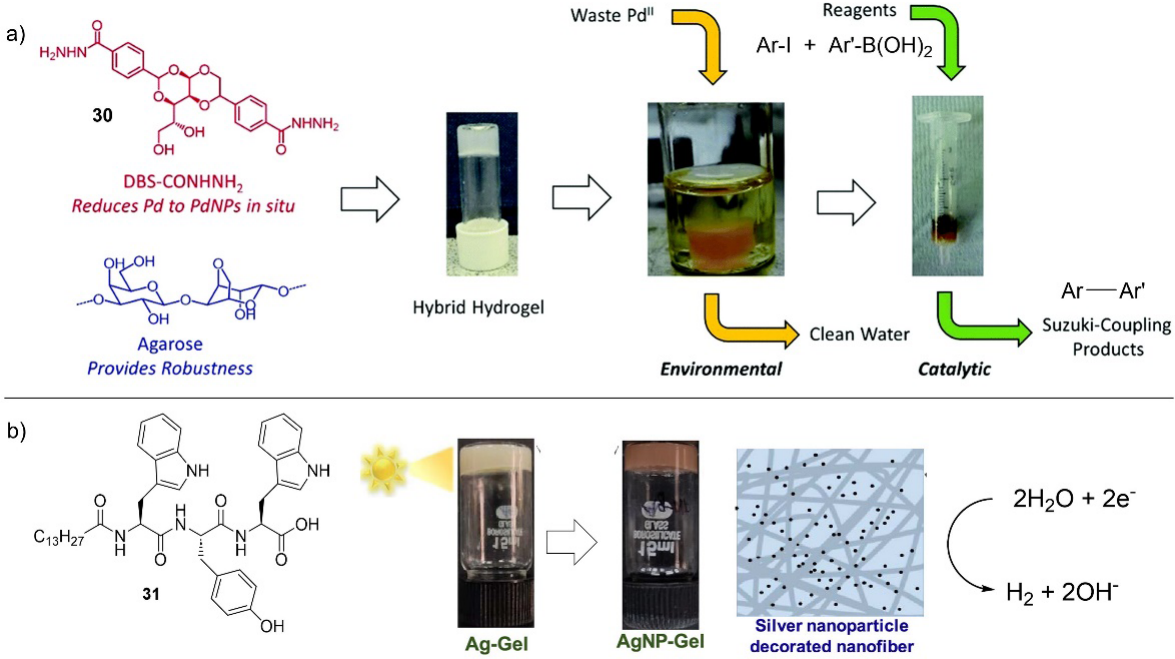
a) LMWG DBS‐CONHNH_2_ **30** mixed with polymer gelator (PG) agarose gives a robust hybrid hydrogel that reduces Pd(II) to ligand‐free PdNPs in situ. The PdNP‐loaded gel is capable of catalyzing Suzuki–Miyaura cross‐coupling reactions in a green and sustainable workflow. Figure reproduced from Reference [107] with permission from the Royal Society of Chemistry. b) LMWG **31** forms gels that are loaded with Ag^+^ ions, which are then converted into AgNPs by UV irradiation. The AgNP‐loaded gel catalyzes hydrogen production from water. Figure adapted from Reference [109] with permission from the American Chemical Society.

Beyond enzymes, it is possible to stabilize other nanoscale catalysts within gel‐phase materials. For example, we have reported gels as providing a unique environment for the formation and stabilization of catalytically‐active ligand‐free precious metal nanoparticles (NPs).^[^ ^106^ ^]^ Our hydrogelator DBS‐CONHNH_2_ (**30**), based on 1,3:2,4‐dibenzylidenesorbitol (DBS), is capable of remediating Pd(II) from water, reducing it to palladium nanoparticles (PdNPs) in situ (Figure 12a).^[^ ^107^ ^]^ The conversion of Pd(II) salts to Pd(0) nanoparticles (NPs) is driven by the oxidation of DBS‐CONHNH_2_ to DBS‐COOH, which retains the gel‐phase properties. The PdNP formation process in the gel occurs in the absence of any standard stabilizing ligands, and as such, the resulting NPs (≈20 nm diameter) can be considered ligand‐free. Normally, ligand‐free metal NPs would not be stable and would be very challenging to work with in ambient conditions as they would aggregate to minimize surface area. We demonstrated that as well as being stable, these PdNP‐loaded gels were catalytically active and could be used in Suzuki–Miyaura cross‐couplings under green and sustainable conditions. Later, we went on to also apply these PdNP‐loaded gels in Heck and Sonogashira cross‐coupling reactions.^[^ ^108^ ^]^

Working with Fairlamb, we explored the performance of these unusual gel‐stabilized PdNPs in mechanistic detail.^[^ ^110^ ^]^ We investigated the arylation of 2,4‐dibromopyridine, a reaction for which the formation of the 4‐substituted product is diagnostic of ligand‐free PdNP catalysis. We indeed found a 10:1 selectivity for the 4‐substituted product over the 2‐substituted product—an excellent level of selectivity. This result confirmed that the gel was indeed stabilizing “naked” PdNPs. Clearly, within the gel, these NPs can be sterically stabilized by the gel network and are generated in stable form without aggregation and with well‐controlled diameters.

There have been other reports of catalytic metal NPs encapsulated within gels, although the speciation of the catalyst has generally been less clearly defined. In early work, Maity and Maitra also reported gels loaded with Pd(II), with PdNPs being formed in this gel on addition of an external reducing agent.^[^ ^111^ ^]^ These gels were also catalytically proficient in Suzuki–Miyaura cross‐coupling reactions, although the mechanism of PdNP surface stabilization was not reported. Gold NPs (AuNPs) formed in gels have been used to catalyze the reduction of 4‐nitrophenol to 4‐aminophenol.^[^ ^112^, ^113^ ^]^ Generally, in these systems, it is argued that the AuNPs are stabilized by direct interactions with the self‐assembled gel nanofibers.

In addition to catalyzing organic reactions, supramolecular gels loaded with metal NPs have also been used as hybrid nanomaterials to catalyze environmentally important reactions. For example. Banerjee and coworkers recently reported a gel loaded with silver nanoparticles (AgNPs) capable of catalyzing the hydrogen evolution reaction (Figure 12b).^[^ ^109^ ^]^ The amphiphilic LMWG (**31**) formed hydrogels in the presence of metal ions such as Ag^+^. On photoreduction, AgNPs were produced in situ within the gel, although the surface stabilization of the AgNPs was not characterized in detail. The AgNP‐loaded gel could catalyze hydrogen production from water with an overpotential of 480 mV at a current density of 10 mA cm^−2^ having a low electrochemical resistance in 0.5 M H_2_SO_4_ electrolyte. The authors highlighted the potential of this system in green energy applications.

Recently, Paul and coworkers used supramolecular gels to stabilize CsPbBr_3_ nanocrystals that usually reuqire stabilization from ligands such as oleyl amine.^[^ ^114^ ^]^ They applied carefully optimized self‐assembling peptides that directly interacted with the CsPbBr_3_ nanocrystals, providing steric protection and enhancing stability in water. Although the primary applications of such nanocrystals are optical, the researchers demonstrated that gel‐stabilized CsPbBr_3_ could photocatalytically degrade dyes such as rhodamine B with minimal lead leaching. However, the direct interactions between the nanocrystals and the gel matrix meant that the product was not released, and the catalyst could therefore not be reused.

Overall, there is significant potential for supramolecular gels to stabilize a variety of nanoscale systems, with the matrix acting as a replacement for the usually required ligands. Both steric effects and direct interactions can stabilize nanosystems within a gel, which can then go on to be applied in synthetic procedures. Such nanocomposites are a promising research direction and further examples of how they can be engineered as smart materials for use in synthesis are given in Section 6.

### 4.2 Stabilizing Highly Reactive Organometallics in Gels

The unique environment within the gel phase suggests the possibility of stabilizing some very highly reactive species by formulating them within a gel. This has the potential to transform the way organic synthesis is done, making highly reactive species much easier and safer to handle, facilitating their transport and storage, and bringing the use of powerful reagents into the hands of nonspecialist researchers.

**Figure 13:**
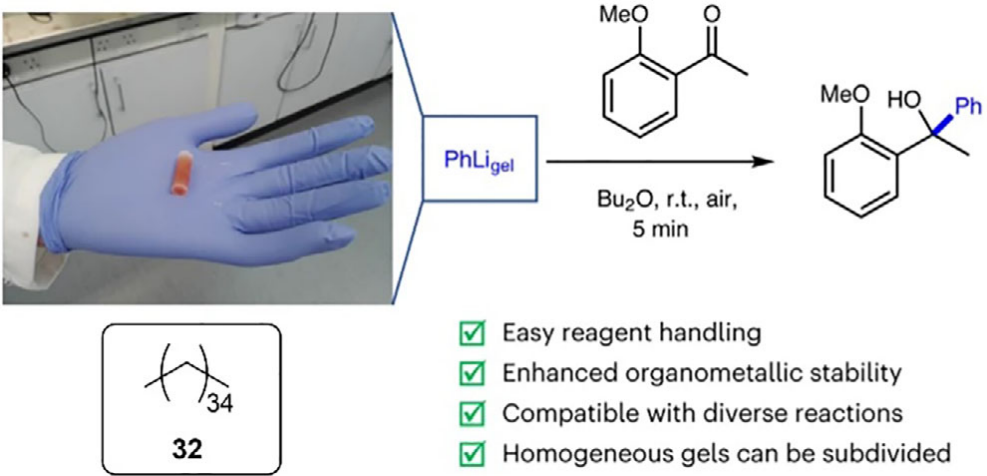
Hexatriacontane (**32**) acts as an LMWG for commercially available organolithium solutions. The photograph shows a gel capsule produced from PhLi being safely handled in the open laboratory. This gel was used for nucleophilic addition into ketones offering a range of advantages. Figure adapted from Reference [115, 116] with permission from Springer Nature.

With the goal of “taming” highly reactive organometallics, working with O'Brien, we set ourselves the ambitious target of attempting to stabilize organolithium reagents so they could be stored, handled, and applied in ambient conditions.^[^ ^115^, ^116^ ^]^ Organolithiums are powerful reagents with widespread synthetic use. Normally, they require careful handling in inert conditions and at low temperatures because of their very low stability. As such, they are inaccessible to nonexpert researchers and even in expert hands have significant costs of use, both in financial and safety terms. To attempt to stabilize such highly basic reagents within a supramolecular gel, we required an LMWG with no acidic groups, and thus selected a long chain alkane (C_36_H_74_, **32**, Figure 13). As first reported by Weiss and coworkers,^[^ ^117^ ^]^ this alkane forms gels in non‐polar organic solvents – we noted organolithium reagents are supplied in such solvents. Pleasingly, we found that gels were formed in a range of organolithium solutions, with the loading of the LMWG controlling stiffness and ease of handling.

Organolithium stability was very significantly enhanced within the gel. For gels made in vials and stored open to air the organolithium retained activity for several hours, after which, solvent evaporation led to some gel cracking and loss of organolithium stability. However, simply placing a lid on the vial prevented evaporation and extended gel stability very significantly. Titration experiments indicated that in a sealed vial, the gelled organolithiums were fully stable for >42 days in ambient conditions. At higher LMWG loadings, more rigid gels were made. In this way, we created gel blocks using a syringe as a mold, which was subsequently cut away to yield a free‐standing gel “capsule”. These gel capsules could be safely handled in the open lab during periods of time required for reaction set‐up (Figure 13) and stored for much longer periods of time in a closed vial. Importantly, recent formal safety testing classified the *n*‐BuLi‐loaded gel as nonpyrophoric and nonexplosive, enabling potential commercial supply of these reagents in gelated form.^[^ ^118^ ^]^ Even if an organolithium gel capsule was dropped in water, much of the organolithium remained intact. Stability to water exposure could be further enhanced, if required, by dipping the gel capsule in melted C_36_H_74_ to form an additional protective layer.

**Figure 14:**
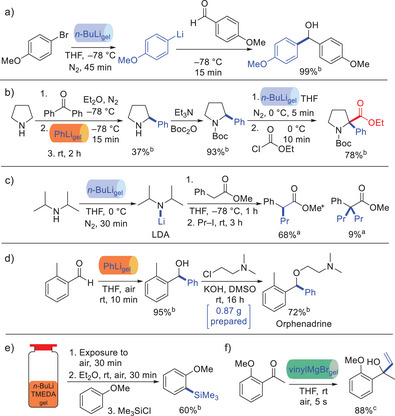
Reactions performed using organometallic gels. a) Bromine–lithium exchange reaction followed by nucleophilic addition of the resulting organolithium into an aldehyde. b) C‐H functionalization in the 2‐position of pyrrolidine, followed by Boc‐protection, then lithiation of the same 2‐position, followed by nucleophilic substitution. c) Synthesis of lithium diisoprpylamide (LDA) from diisopropylamine followed by its use as a strong base to substitute the α‐position of an ester. d) Gram‐scale synthesis of orphenadrine based on nucleophilic addition of phenyl lithium into a ketone followed by nucleophilic substitution of the resulting alcohol into an alkyl halide. e) Formulation of organolithium gel in a vial in the presence of tetramethylethylenediamine (TMEDA) and use of this gel in an ortho‐lithiation reaction, trapped by trimethylsilyl chloride (TMSCl). f) Use of gel incorporating vinyl magnesium bromide for nucleophilic addition into a ketone.

We performed a wide variety of reactions with these gels in ambient conditions, for example, nucleophilic additions (Figure 13), bromine–lithium exchange (Figure 14a), and C–H functionalization (Figure 14b). In general, the gels were broken down on stirring, releasing the organolithium. To demonstrate wider potential, we used the BuLi organogel to synthesize lithium diisopropylamide (LDA), which was then used as a strong base in subsequent reactions (Figure 14c). We used PhLi organogel in a gram‐scale synthesis of the muscle relaxant orphenadrine, a key drug target (Figure 14d). It was also demonstrated that ligands such as tetramethylethylenediamine (TMEDA) can be incorporated in the gel alongside the organolithium. This activated the organolithium and required higher LMWG loading within a vial for stability but the resulting gel was proficient in ortho‐lithiations (Figure 14e). Finally, in preliminary studies, we successfully applied this technology to organomagnesium reagents (Figure 14f).

Formulating organolithiums in gels has significant advantages over other possible protective formulations, such as liquid‐filled capsules,^[^ ^119^, ^120^, ^121^, ^122^ ^]^ as it yields a material with a homogeneous loading of the reactive species. This makes bulk manufacture potentially very straightforward, with subdivision of the resulting bulk gels giving rise to smaller gel blocks with reproducible amounts of the organolithium. Indeed, we demonstrated that cutting one of our gel blocks into portions led to predictable organolithium doses. These gels enable organolithium‐mediated reactions to be performed in ambient conditions with no unusual safety precautions, which has the potential to transform lab‐scale chemistry. Currently, reagents such as organolithiums are highly regulated and have a high burden of safety and environmental costs. Simple, low‐cost LMWG additives such as these have the potential to remove some of the hurdles to organolithium use and open them up to a much wider range of end users. Furthermore, this approach has the potential to change the way such reactive species are transported and stored by the chemicals industry.

**Figure 15:**
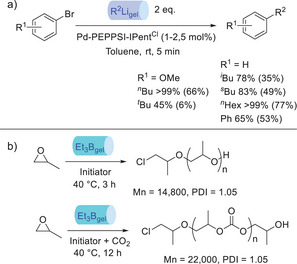
a) Use of hexatriacontane‐based gels for the palladium‐catalyzed cross‐coupling of an aryl bromide with a variety of stabilized organolithium reagents. b) Hexatriacontane gel‐stabilized organoborane reagents for the polymerization of propylene oxide in the absence (top) and presence (bottom) of CO_2_.

Feringa and Visser used this organolithium gel technology in their palladium‐catalyzed cross‐coupling methodology in which an organolithium is reacted with an aryl bromide (Figure 15a).^[^ ^123^ ^]^ They noted the very significant advantages of the gel formulation, highlighting the ability to perform reactions in 5 min at room temperature, eliminating the previously required slow addition and strict use of inert atmosphere. Indeed, they commented that these organolithium gels “tremendously increased process safety”, as illustrated by performing a gram‐scale reaction to produce one of the photochromic switches widely exploited by the Feringa group without any extraordinary safety precautions. Pleasingly, they also prepared organogels containing *t*‐BuLi. These gels primarily gave dehalogenation products, so were not optimal in this specific case, but their work demonstrated the capacity for this encapsulation technology to be applied to the most reactive and most hazardous organolithium reagents.

In further development of this technology, Chidara et al. reported the encapsulation of highly unstable boranes and borinanes within the same type of organogel.^[^ ^124^ ^]^ Specifically, they encapsulated triethylborane in C_36_H_74_ gel block and demonstrated that it could be handled in the open laboratory without need for a glovebox. The researchers found the organogel was somewhat brittle, and although stability was significantly enhanced over free solution, this led to some porosity, which allowed oxygen to slowly deactivate the organoborane. They, therefore, preferred to dip their organogel blocks in melted C_36_H_74_ so the surface was covered with an additional thin protective layer. These systems retained 99% active boron even up to 4 weeks. On heating the gel blocks, organoboranes could be freed from the organogel and were used to mediate the homo‐polymerization of propylene oxide (PO) and its copolymerization with CO_2_ (Figure 15b). It was reported that 92% of the LMWG could be recovered from the reaction using a simple hexane washing procedure. The authors, therefore, concluded this was a promising approach to enhance the stability and use of borane compounds.

## 5 Reactions in Supramolecular Gels

### 5.1 Organizing LMWGs for Controlled Reactions

**Figure 16:**
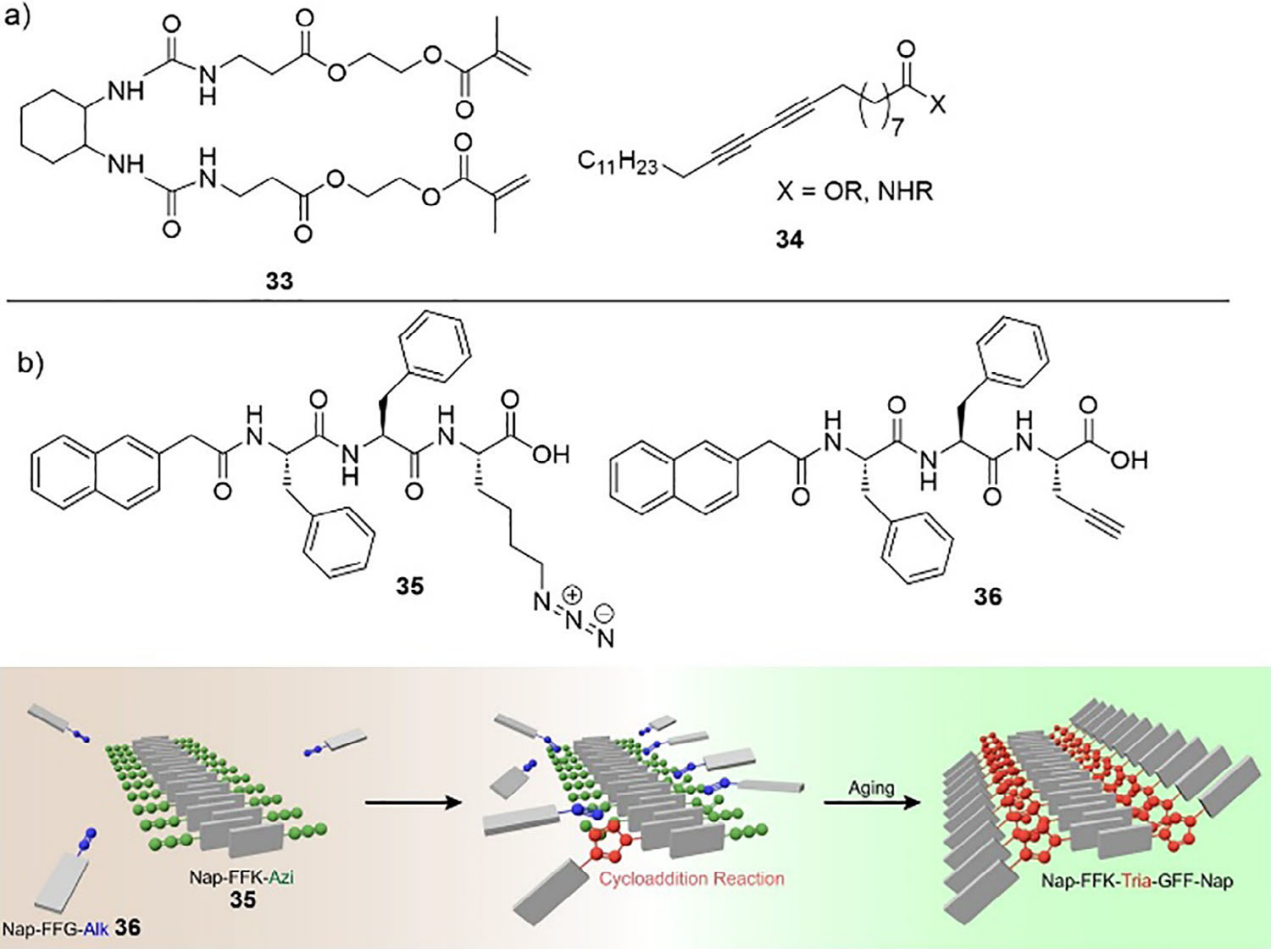
a) LMWG **33** modified with acrylates and LMWG **34** incorporating a bis‐alkyne are both examples of molecules that are capable of undergoing effective polymerization in a self‐organized gel phase. b) Coassembly of azide‐modified LMWG **35** and alkyne‐modified LMWG **36** enables the click chemistry reaction between them, even in the absence of a metal catalyst. Figure adapted from Reference [128] with permission of Springer Nature.

Even in the absence of a catalyst or reagent, supramolecular gels can offer a unique environment in which reactions can take place with different outcomes. Indeed, a self‐assembled gel can be considered a unique “reactor” for synthetic chemistry. There has been considerable interest in reactions of the LMWG itself, such as cross‐linking between LMWGs or modifying the LMWG structure in situ within a gel. In work from the late 1990s and early 2000s, a variety of research groups noted that in the gel phase, functional groups were brought into close proximity, facilitating their mutual reaction. For example, LMWGs that had polymerizable groups, like acrylates (e.g., **33**) or bis‐alkynes (e.g., **34**), underwent “solid‐state” polymerization reactions (Figure 16a).^[^ ^125^, ^126^, ^127^ ^]^

In 2006, the Díaz group used click chemistry to cross‐link self‐assembled LMWGs modified with peripheral azide and alkyne groups, significantly increasing the mechanical strength of the gel.^[^ ^129^, ^130^ ^]^ In recent eye‐catching work, Liang and coworkers reported that coassembled LMWGs modified with an azide (**35**) and an alkyne (**36**) could undergo “click” coupling between them with excellent regioselectivity, even in the absence of a metal catalyst (Figure 16b).^[^ ^128^ ^]^ They proposed this was a result of the preorganization of reactive groups imposed by self‐assembly.

**Figure 17:**
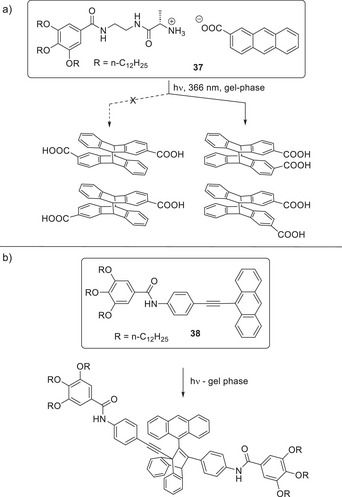
a) Two‐component acid‐amine organogelator **37** undergoes photo‐induced dimerization in the gel phase with selectivity for head‐to‐head products over head‐to‐tail products. b) LMWG **38** undergoes photodimerization to give >90% of the unusual 4+2 cycloaddition product in the gel phase, whereas in solution, a 4+4 product (not shown) is formed.

In 2009, Dawn and coworkers described a two‐component organogelator **37** in which one of the two components, anthracene carboxylate, was able to undergo photoinduced dimerization in a controlled way as a result of its confinement within the self‐assembled gel fibers (Figure 17a).^[^ ^131^ ^]^ Interestingly, they found that in the gel, only *anti* and *syn* head‐to‐head products were obtained, with no evidence of any head‐to‐tail products. This clearly indicates the excellent fidelity in photochemical reactions that can be obtained by organizing a reagent using a nanostructured gel network. In later work, Ajayaghosh and coworkers investigated LMWG **38**, which incorporates 9‐phenylethynylanthracene within its structure, and demonstrated that photocyclization reactions performed in its gels gave rise to products that would otherwise be hard to obtain (Figure 17b).^[^ ^132^ ^]^ Specifically, the [4+2] photoadduct was obtained in >90% yield in preference over the [4+4] products that are observed in the solution phase.

However, in the selected examples briefly described above, the LMWG itself is reacting in a controlled manner—such gels are not typically able perform general organic reactions as they are quite specific to the design of the LMWG itself, that must have the capacity both to self‐assemble and then subsequently react in a controlled way. In contrast, there is emerging interest in the use of gels as more general nanoreactors, not as a result of being catalytically active or containing embedded active reagents (see above and Sections 2, 3, 4). Such gels can provide unique confined microenvironments that influence reaction outcomes as described in the next section.

### 5.2 Gels as Nanoreactors

**Figure 18:**
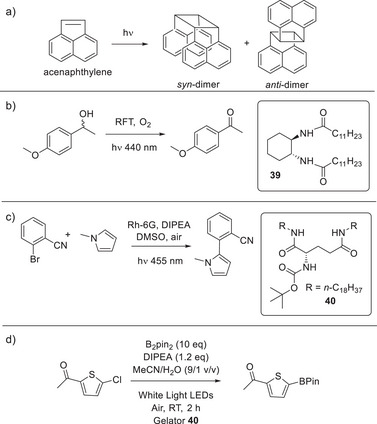
a) Dimerization of acenaphthalene to give *syn* or *anti* products—the selectivity of this reaction is modified by a variety of bile acid–based LMWGs. b) LMWG **39** promotes photo‐oxidation of 1‐(4‐methoxyphenyl)ethanol catalyzed by riboflavin tetraacetate (RFT), even in the absence of a proton transfer agent. c) Photomediated coupling between an aryl bromide and *N*‐methylpyrrole mediated by rhodamine‐6G (Rh‐6G) with diisopropylethylamine (DIPEA) in dimsethylsulfoxide (DMSO), performed in a supramolecular gel based on LMWG **40**, provides protection against the adverse effects of oxygen on the excited state of the catalyst. d) The photo‐induced borylation of an aryl halide with bis(pinacolato)diboron (B_2_pin_2_) is facilitated by being performed within the network of a supramolecular gel based on **40**.

Perhaps the earliest work using a supramolecular gel as a general “nanoreactor” was from Bhat and Maitra in 2007, who demonstrated that photodimerization reactions could proceed in gels with higher efficiencies and different selectivities (Figure 18a).^[^ ^133^ ^]^ They used a variety of gels based on bile acid LMWGs with different structures to control the dimerization of acenaphthalene, giving either *syn* or *anti* dimer products. The native reaction tends to have a slight preference for the *syn* product, but when performed within the supramolecular gel network, the system favored the *anti* product, with up to 5:1 *anti*/*syn* selectivity. Interestingly, stiffer, more rigid gels appeared to have a larger effect on the product ratio.

Díaz and coworkers have more recently pioneered the investigation of photoreduction and photooxidation reactions performed in supramolecular gels. In 2013, they studied the photo‐oxidation of 1‐(4‐methoxyphenyl)ethanol catalyzed by riboflavin tetraacetate (RFT) (Figure 18b).^[^ ^134^ ^]^ They found that simple physical incorporation of the reagents within a variety of organogels enhanced the reaction. In particular, the organogel based on a cyclohexane‐based bis‐amide LMWG (**39**) in CH_3_CN achieved full conversion in 60 min, even in the absence of thiourea, which is normally required as a proton transfer agent. It was suggested that the excellent gel‐induced performance indicated interactions between the gel fibers and substrate or catalyst. Intriguingly, it was demonstrated that the precise nanoscale morphology impacted on the reaction outcomes, with stiff, highly entangled networks usually being associated with lower reaction rates, whereas wrinkled, laminated morphologies seemed to be associated with enhanced reactivity.

Díaz and coworkers later went on to study the photoreduction of aryl halides in air using a supramolecular gel containing a photosensitizer/emitter pair in aerobic conditions.^[^ ^135^ ^]^ They noted that when the reaction was performed in solution, the pink color of the original solution was lost as the excited state was quenched by oxygen. However, in the gel phase, there was no change in color, indicating that the excited state was stabilized in standard aerobic conditions. This hinted that the gel environment could protect the reaction intermediate against the adverse effects of oxygen. Díaz and coworkers built on this observation by showing that air‐sensitive photoredox chemistry can be performed in a self‐assembled gel under aerobic conditions as a result of reaction confinement and the blockage of oxygen diffusion.^[^ ^136^ ^]^ Specifically, they functionalized aryl halides using rhodamine‐6G as a photocatalyst in aerobic conditions, with the network formed by LMWG **40** preventing quenching of the excited state of the active catalyst and thus ensuring success of the reaction (Figure 18c). For the test coupling of 2‐bromobenzonitrile with *N*‐methylpyrrole, the yield improved from just 5% in solution to 53% within the gel. Indeed, the authors found the gel‐phase system performed as well in aerobic conditions as conventional solution phase chemistry under strictly inert conditions. Clearly, therefore the unique environment in a gel can protect reactive intermediates within the network. This relates to the work in Section 4, in which it was shown that highly reactive catalysts or reagents that would not usually be stable in ambient conditions can be stabilized within simple self‐assembled gels.

In later work, Pérez‐Ruiz and coworkers demonstrated that borylation of heteroarenes could be achieved within a supramolecular gel “nanoreactor” based on LMWG **40** (Figure 18d).^[^ ^137^ ^]^ Visible light from white light‐emitting diodes (LEDs) was used as the energy source for this photocatalyst‐free radical reaction. Once again, the ability of the gel to protect reactive intermediates from oxygen played a role in making this reaction work effectively within the organogel, but not in free solution, and also accelerated the reaction.

Moving away from photochemistry, Haldar and coworkers reported a pentapeptide gel, which they applied in halogenations, Diels–Alder reactions, and the Morita–Baylis–Hillman (MBH) reaction.^[^ ^138^ ^]^ The halogenation of aniline and the Diels–Alder reaction between furan and maleic anhydride increased 2‐fold in rate, whereas the MBH reaction between benzaldehyde and ethyl acrylate increased 1.5‐fold in the peptide nanoreactor. Structural analysis of the gel led the authors to suggest it had π‐rich channels that may act as a platform for promoting these organic reactions.

Clearly, there is considerable further scope for reactions to be performed in gels with a variety of potential impacts on reaction outcomes. Although it is clear that gels have particular use in photo‐induced reactions and can provide protective microenvironments, the design principles of gels to mediate different classes of reaction have not yet emerged. In future work, it will be important to try and generate a more systematic understanding of the ways LMWG structure, nanoscale morphology, and other aspects of assembly, control the outcomes of different reations. With the emergence of a structure‐activity based understanding of gels as nanoreactors, it is possible that a range of LMWGs could produce a toolkit of gels with synthetic applications. It will also become possible to use gel nanoreactors that incorporate other active species or active LMWGs in order to gain holistic control over multistep reactions.

## 6 Engineering Supramolecular Gels to Harness or Enhance Reactivity

Beyond simply making a catalytic/reactive gel and using it in synthesis, it is important to think about the way the gel is formulated so that it can be delivered into the reaction in the most useful and effective way. Attempts to transform the ways in which chemical reactions are done in the laboratory are a vibrant theme in modern chemical research.^[^ ^139^, ^140^, ^141^ ^]^ Indeed, disrupting traditional approaches to synthesis opens the possibility of a greater degree of automation, better synthetic workflows, safer processes, and easier product purification. Ultimately, it may be possible to achieve much of chemical synthesis using simple kits or automated systems, suitable for nonspecialist researchers, or even the end‐users of the desired products. As such, chemical engineering of reaction processes has the potential to be a truly disruptive technology. Supramolecular gels could play a significant role in this process, as described in Sections 6.1, 6.2, 6.3, 6.4.

### 6.1 Molding and Reinforcing Supramolecular Gels

One of the simplest ways to use gels in synthesis is to ensure they can be easily handled and manipulated, with an easy way of achieving this being molding them into desired shapes.

This was exemplified in our description of organolithium gels in Section 4.2, in which we noted that organolithium gels could be created in vials and reagents can be added to the top, or they could alternatively be formulated at higher loading as self‐standing gel blocks (Figure 13). These blocks were fabricated by molding the supramolecular gel in a surrounding vessel, then cutting the mold away. Notably, a high loading of LMWG was required to achieve self‐standing organolithium gels, reflecting the relatively weak/soft nature of supramolecular gels (see below). Organolithium gel blocks could be easily handled in the laboratory, and/or subdivided into portions and subsequently added into reactions.^[^ ^112^, ^113^ ^]^ Gel “engineering” can therefore be a key step in translating gel‐encapsulated reaction technologies into real‐world applications.

Also taking a molding approach, Marr and coworkers made use of a silicone mold to shape their relatively robust enzyme‐loaded ionic liquid gels (described in Section 3.1) in order to fabricate gel beads for easy use in catalysis.^[^ ^88^ ^]^ They used molds that could produce gel beads with different sizes and noted that the smaller beads had higher activities, likely resulting from their higher surface areas. The catalytic enzyme‐loaded ionogel beads could be used >10 times in reactions without loss of activity, although the smaller beads showed greater run‐to‐run variation. The use of easily‐handled gel beads facilitate the addition and removal of the gel from the reaction vessel.

In the cases above, the rheological properties of the gels were sufficient to enable self‐standing gels or could be optimized to achieve this by increasing LMWG loading. In many cases, however, supramolecular gels will not readily form stable self‐standing objects. In such cases, an alternative approach is to reinforce a supramolecular gel by coformulating the LMWG with an additive, chosen for its rheological performance, that is capable of reinforcing the system. Polymer gelators (PGs) are particularly useful in this regard; indeed the combination of LMWGs with PGs to combine LMWG activity with PG rheological performance is an increasingly widely applied strategy.^[^ ^142^, ^143^, ^144^ ^]^

**Figure 19:**
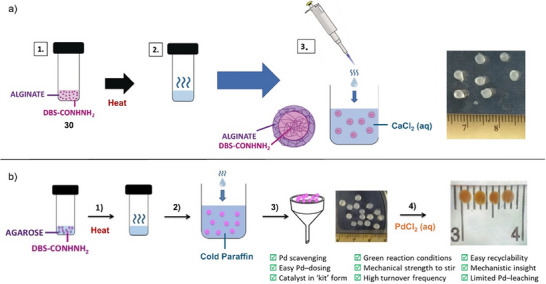
a) Formation of gel beads by dropwise addition of a hot solution of LMWG DBSCONHNH_2_ (**30**) and alginic acid into aqueous calcium chloride. The photograph shows gel beads with diameter ≈3 mm. Image reproduced from Reference [145] with permission of Wiley‐VCH. b) Formation of gel beads by dropwise addition of a hot solution of LMWG DBS‐CONHNH_2_ (**30**) and PG agarose into cold paraffin. After filtration and washing, the beads are loaded with PdNPs by soaking in aqueous Pd(II). The photograph shows PdNP‐loaded gel beads with diameter ≈1.8 mm. Image reproduced and adapted from References [146] and [110].

Having uncovered the potential of PdNP‐loaded supramolecular gels based on DBS‐CONHNH_2_ (**30**) in cross‐coupling reactions (see Section 4.1), we wanted to engineer these materials so that the “naked” PdNPs in the gel could be more easily dosed into reactions and potentially used in “kit form” by nonspecialist researchers. To reinforce the gels, we initially combined the LMWG with a calcium alginate polymer gel (Figure 19a).^[^ ^145^ ^]^ On adding a hot solution of DBS‐CONHNH_2_ and alginic acid dropwise into aqueous calcium chloride, calcium alginate rapidly formed a polymer gel “shell” within which the LMWG could then slowly assemble as the system cooled, yielding a core–shell multicomponent gel bead. The bead diameter was controlled by the droplet size on addition. These hybrid gel beads reduced PdCl_2_ producing PdNPs in situ— as such, the LMWG plays the expected active chemical role. The PdNP‐loaded beads could be easily added to Suzuki–Miyaura cross‐coupling reactions with easy reaction dosing, therefore the calcium alginate rheologically reinforces the system and stabilizes the gel beads. However, these first‐generation beads were quite soft, and it was difficult to vigorously stir the reactions or recycle the gel beads after use.

**Figure 20:**
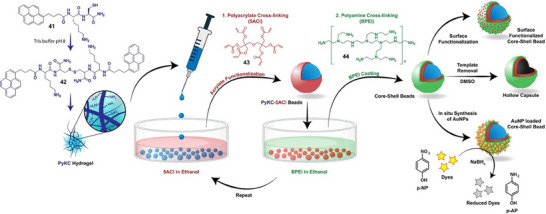
Thiol precursor **41** is converted to disulfide LMWG **42**, which can be extruded from a syringe into ethanol forming gel beads. To further stabilize the beads, polyacrylate **43** was added to the ethanol and is adsorbed onto the bead surface. Placing the modified bead in an ethanol bath containing branched poly(ethyleneimine) (BPEI) **44** then cross‐links the bead surface through Michael addition. This process can be repeated to give multilayer coverage of the gel bead surface. Synthesis of AuNPs on the bead surface mediated by the presence of **44** creates a hybrid AuNP‐loaded gel bead that can be used to reduce nitroaromatics and azo dyes. The LMWG template can also be removed from the core of the bead to fabricate hollow capsules, or the surface functionalized in other ways. Figure reproduced from Reference [147] with permission from Elsevier.

We, therefore, optimized the engineering of this system, replacing the calcium alginate PG with agarose (Figure 19b) and using cold paraffin to trigger the formation of both LMWG and PG networks at the same time.^[^ ^110^ ^]^ This creates interpenetrated hybrid PG/LMWG gel beads, with both networks being formed simultaneously on cooling. They were much more robust and easily handled than the beads formed with calcium alginate. As such, the agarose‐stabilized PdNP‐loaded gel beads had excellent recyclability and reusability, offering a sustainable and easily‐handled approach to using otherwise unstable “naked” PdNPs in synthetic chemistry. Furthermore, leaching of PdNPs from the gel was very low, with catalysis occurring solely within the gel. We consider this LMWG/PG gel‐mediated approach to be optimal for creating “reaction kits” for the easy application of ligand‐free PdNPs in synthetic chemistry.

Das and coworkers employed an extrusion approach to create catalytic beads based on a peptide LMWG (Figure 20).^[^ ^147^ ^]^ In this case, the hydrogel based on disulfide **42** was capable of being injected through a needle, with a 5% wt/vol sample being extruded into ethanol. During injection, the gel was broken down into sol droplets, and then rapidly reformed, solidifying as a bead. This was facilitated by the high LMWG loading being applied but also the fact that the insoluble self‐assembling disulfide **42** can equilibrate with the water‐soluble thiol precursor **41**. Although these beads were relatively stable, they could be significantly further stabilized by the formation of a cross‐linked surface coating. This was achieved by having dipentaerythritol hexa‐acrylate (**43**) present in the ethanol. This additive coats the surface of the bead, which was then placed in an ethanol bath containing branched poly(ethylene imine) (**44**). This polymer reacts with the preadsorbed acrylate, leading to cross‐linking of the surface shell via multiple Michael addition reactions. This coating cycle could be repeated multiple times to build up a stable multilayer shell on the bead. Once the process was complete, AuNPs were synthesized on the beads in situ as a result of the innate ability of poly(ethyleneimine) to reduce Au(III) to Au(0). The resulting AuNP‐decorated core–shell beads were used for the reduction of nitroaromatics and azo dyes, over several catalytic cycles with no loss of activity. The authors noted that this gel bead approach facilitated the handling, storage, and stability of AuNPs during repeated use and over long periods of time. In this case, the key role played by the LMWG is primarily thixotropic, in terms of its ability to form the bead on injection. However, the reversible self‐assembled nature of the supramolecular gel bead core also meant that the use of a good solvent (such as dimethylformamide or dimethylsulfoxide) was able to remove significant amounts of LMWG from the beads, leaving hollow cross‐linked capsules based on the cross‐linked polymer shell—such systems may also have future applications.

Very recently, Das and coworkers grew gold nanostars in situ on the surface of this type of core–shell hydrogel bead and demonstrated that the hybrid materials exhibited oxidase‐like activity when exposed to light.^[^ ^148^ ^]^ Photoexcitation of the gold nanostars generates singlet oxygen through the interaction of positive holes and superoxide radicals, leading to the photo‐oxidation of 3,3′,5,5′‐tetramethylbenzidine (TMB). This system was used to detect uric acid, which reduces the oxidized colored form of TMB. The authors noted that the gold nanostars retained catalytic activity on these hybrid materials for extended periods of time (e.g., >60 days incubated in aqueous media), over multiple cycles and under stirred conditions.

LMWGs evidently have great potential in the fabrication of shaped materials for use in synthesis, enabling easy dosing into reactions. Combining LMWGs with other materials to create multicomponent hybrid systems, in which each component brings its own characteristics to the overall hybrid, has particular power. It is expected that in the future, one direction of research may see the development of smart gels capable of promoting multistep reaction sequences, perhaps using a layer‐by‐layer approach to gel fabrication. Such materials, capable of dispensing reagents in a stepwise manner as the gel disassembles would potentially be able to telescope multiple reaction processes and allow complex chemical interconversions to be performed with the simple addition of a single gel bead.

### 6.2 Microstructured Supramolecular Gels in Catalysis

Beyond the formation of macroscopic gel beads that can be physically handled, added to reactions, and/or removed, as described above, it is possible to consider making such systems smaller (e.g., microscale). This opens up a range of possibilities for the use of supramolecular gels in reaction engineering.

For example, Das and coworkers mixed their cytochrome c‐loaded hydrogel described in Section 3.^[^ ^88^ ^]^ with a nongelating surfactant amphiphile. This disintegrated their gel into smaller surfactant‐stabilized particles (≈1 µm diameter). These gel microparticles showed a remarkable enhancement in activity of 675‐fold for the H_2_O_2_‐mediated oxidation of pyrogallol, approximately double that of the bulk gels. It was reasoned that this enhancement may occur as a result of the gel microparticles having larger surface areas and/or the surfactant complementing the mass‐transfer process at the amphiphilic fibers by assisting in the formation of larger interfacial domains in the overall supramolecular structure.

**Figure 21:**
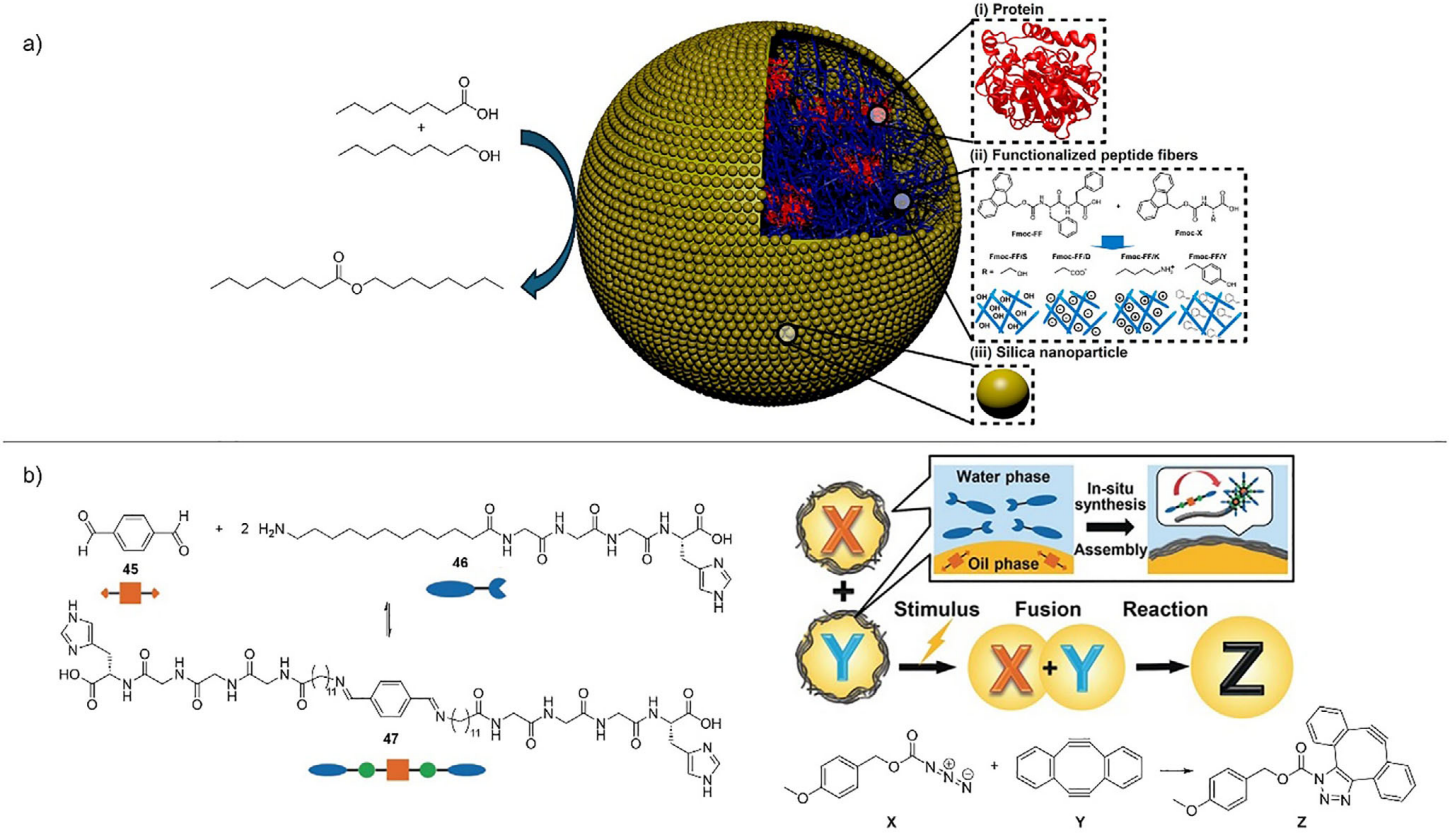
a) Microparticles of Fmoc‐FF (**29**) and Fmoc‐X two‐component LMWG, loaded with *Candida Antarctica* (CalB) and with surface stabilized with hydrophobic silica nanoparticles form a gelled emulsion that catalyzes the esterification of octanoic acid and octanol in hexane. Figure adapted from Reference [149] with permission from the American Chemical Society. b) Oil‐soluble bis‐aldehyde **45** and water‐soluble amine **46** react dynamically at the droplet interface of an oil‐in‐water emulsion to form bis‐imine **47** that acts as an LMWG at the surface of the droplet. Droplets can then be fused by controlling temperature and pH. If orthogonal droplets are loaded with an azide (**X**) and a strained alkyne (**Y**), then droplet fusion can give rise to controlled reaction between them to yield product **Z**. Figure adapted from Reference [150] with permission from Wiley‐VCH.

Ulijn and coworkers used hydrophobically‐modified SiO_2_ nanoparticles to emulsify aqueous droplets of LMWG precursors incoporating Fmoc‐FF (**29**) in heptane, loaded with lipase B from *Candida Antarctica* (CalB) (Figure 21a).^[^ ^149^ ^]^ The resulting gelled particles in the Pickering emulsion had an average diameter of ≈8 µm and were assembled from a variety of two‐component LMWGs (Fmoc‐FF/Fmoc‐D, Fmoc‐FF/Fmoc‐K, Fmoc‐FF/Fmoc‐S, and Fmoc‐FF/Fmoc‐Y). The catalytic performance of these CalB‐loaded microparticles in the esterification of octanol and octanoic acid was tested in hexane by introducing the substrates into the emulsion. The gelled emulsions showed significant improvements in activity compared with native CalB in a liquid biphasic system. The largest enhancement (3.9‐fold) was observed for Fmoc‐FF/Fmoc‐S, which is the most hydrophilic of the LMWGs. This led the authors to suggest that the enzyme could diffuse more freely through the more hydrophilic LMWG networks and better access the biphasic emulsion interface where catalysis takes place. The emulsions could be removed by centrifugation and reused over four cycles, demonstrating that the gel phase can still allow for recyclability even when the particle size is very small.

Murayama and coworkers also employed an interfacial approach toward microdroplet fabrication and then used these microdroplets to control reactivity (Figure 21b).^[^ ^150^ ^]^ The LMWG was synthesised in situ at an oil–water interface via reaction between an oil‐soluble aldehyde (**45**) and a water‐soluble amine (**46**). This led to bis‐imine LMWG **47** that formed a gelator shell at the surface of the oil microdroplets, stabilizing them. Fusion between droplets could be achieved by the addition of HCl and heating to 80 °C, as visualised using microgel droplets loaded with different dyes. Orthogonal gel‐stabilized microdroplets were loaded with either an azide (**X**) or a strained alkyne (**Y**). Only on microdroplet fusion thermally triggered by LMWG disassembly did the click reaction between components to form the product triazole (**Z**) take place, demonstrating the potential of microgels to encapsulate reagents and hence control their reactivity as a result of this spatial control.

The potential of microsized systems such as emulsions, to increase interfacial surface area between different phases, can have significant beneficial effects on mass transport that can be important in the engineering of a variety of chemical reaction processes. The combination of surfactants and gelators is also an area of considerable fundamental physicochemical interest.^[^ ^151^ ^]^ The disadvantage of this approach is that it can be difficult to separate products from microscale assemblies without resorting to techniques such as dialysis or centrifugation, which are less popular in standard synthetic workflows. Currently, the use of microscale gel systems in synthetic chemistry remains relatively unexplored; however, combined with the surge in interets in supramolecular gels as functional protocell systems (see Section 2.4), which also have microscale sizes and can control reactivity in a spatially‐resolved manner, it seems very likely that this area of research will rapidly develop.

### 6.3 Supramolecular Gels in Flow Chemistry

The high degree of compatibility between gels and the solvent phase, opens the possibility of using such systems as a “solid‐like” phase within a flow reaction setup. Flow chemistry offers the potential to easily isolate pure products, separate from reagents and catalysts, as a result of the device design. Furthermore, the automation possible in such systems is highly desirable in modern workflows for synthetic chemistry.^[^ ^152^, ^153^ ^]^

**Figure 22:**
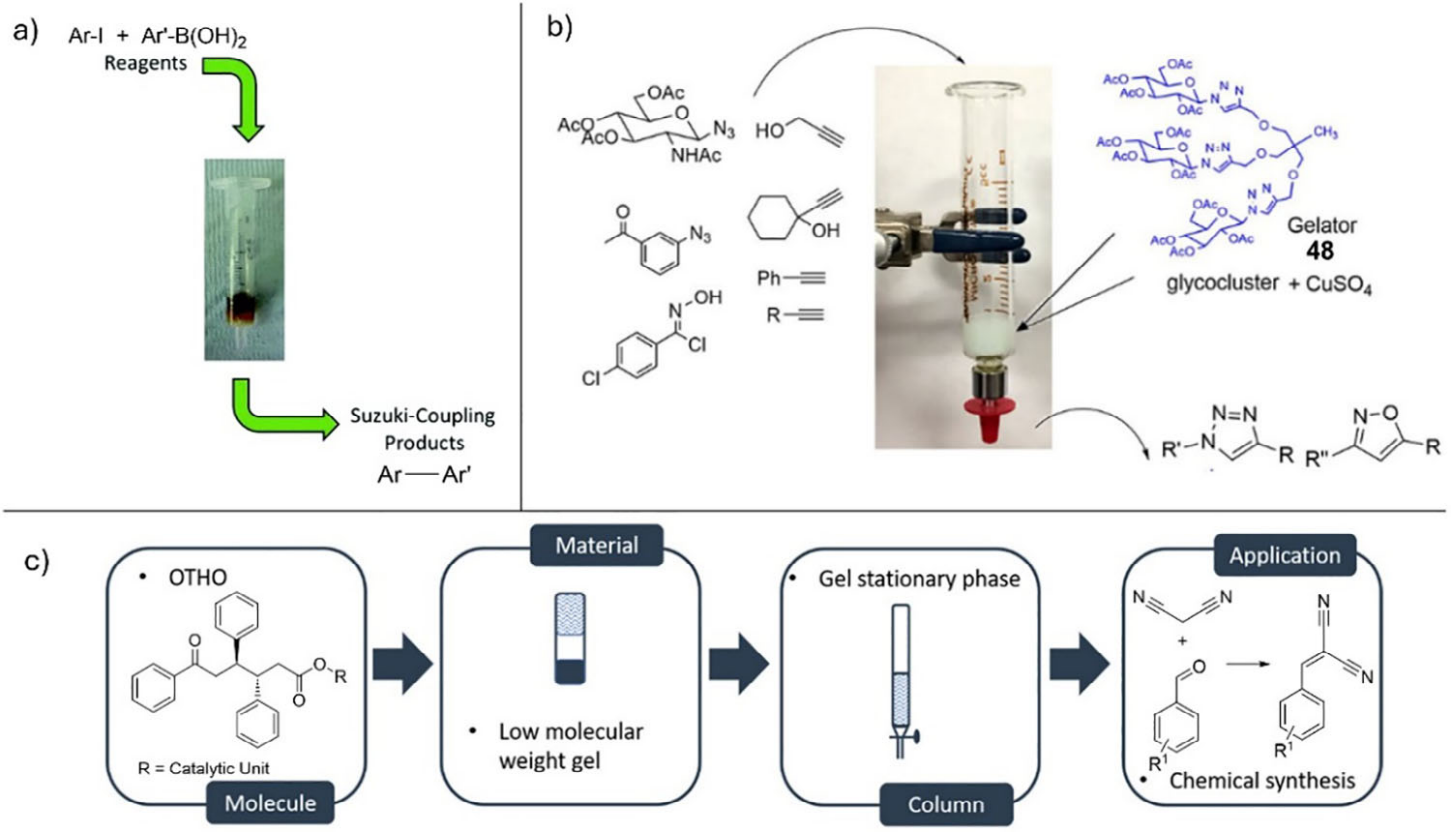
a) Simple gravity‐driven flow through a PdNP‐loaded gel in a plastic syringe led to quantitative conversion in the cross‐coupling of aryl iodides and aryl boronic acids to give bis‐aryl products, which were easily isolated in pure form. Figure adapted from Reference [107] with permission from the Royal Society of Chemistry. b) Triazole‐functionalized glycoclusters can from supramolecular gels and bind copper ions, acting as catalysts for the click reaction—by optimizing the choice of glycoclusters, a suitable gel for packing into a column was found and flow‐through click reactions between a variety of azides and alkynes were performed. Figure adapted from Reference [154] with permission from the American Chemical Society. c) The oxotriphenylhexanoate (OTHO) scaffold functionalized with a catalytic DABCO unit (R) forms a supramolecular gel that can be used in flow‐through catalysis of the Knoevenagel reaction between malonitrile and a substituted benzaldehyde. Figure reproduced from Reference [155] with permission from the American Chemical Society.

As a simple example, we demonstrated the potential of using catalytic supramolecular gels in flow when applying our PdNP‐loaded supramolecular gels to perform Suzuki–Miyaura cross‐couplings (Figure 22a).^[^ ^107^ ^]^ We formed the gel in a column made from a plastic syringe with cotton wool in the exit using a heat–cool approach. PdNPs were loaded in situ and then we performed a gravity‐driven cross‐coupling between a variety of aryl iodides and phenylboronic acid. The relatively short retention times forced us to use a stronger base (KOH instead of K_2_CO_3_), and the reaction was performed in an incubator at 50 °C. In flow reactors, solubility of all components is of key importance—this meant we had to change solvent in some cases from EtOH/H_2_O to poly(ethyleneglycol) (PEG) 200. Flow rates were typically slow but quantitative conversions were achieved and pure products easily isolated.

Wang and coworkers developed a flow reactor capable of performing copper‐catalyzed azide/alkyne cycloaddition (“click”) reactions (Figure 22b).^[^ ^154^ ^]^ Their gelator was based on a triazole‐functionalized glycocluster **48** that was capable, in solution, of binding copper ions and hence acting as a ligand for “click” reactions. To formulate their active system, they coassembled glycoclusters that formed effective gels, with others that were more effective catalysts, finding that in the gel phase, reactions were considerably faster than in the control mixture. They used these systems to form gel columns. This required considerable screening, but ultimately yielded a system that could perform “click” reactions between phenylacetylene and an aryl azide in ethanol/water with quantitative conversion. The process could be repeated six times, with an overall yield of 93% after washing the column with ethanol to ensure all product was eluted. Products were obtained in pure form because the catalysts were immobilized in the solid‐like phase of supramolecular gel column. A gel column with a different composition was also fabricated to catalyze the reaction between sugar azides and propargyl alcohol.

Sundén and coworkers developed LMWG **49** for flow‐through catalysis of the Knoevenagel reaction (Figure 22c).^[^ ^155^ ^]^ This LMWG was based on an oxotriphenylhexanoate (OTHO) scaffold that was functionalized with a catalytically‐active 1,4‐diazabicyclo[2.2.2]octane (DABCO) unit. Tertiary amines, such as DABCO, are known to catalyze the Knoevenagel reaction. This LMWG formed stable gels in a range of organic solvents, which were considered useful for organic synthesis, with i‐PrOH being selected. The gelator was dissolved in i‐PrOH (≈1% wt/vol), heated, loaded into a column, and then allowed to cool. Gelation was triggered via sonication and the resulting gel washed with i‐PrOH before use. The substrates (malonitrile and 4‐substituted benzaldehyde) were allowed to diffuse into the gravity‐driven column, where they were retained for 60 min. The column was subsequently eluted with EtOAc to release the product. All reactions proceeded with full conversion and isolated yields of 65%–70%. The gel could be reused five times, with the major contributor to loss of activity being product accumulation in the gel.

**Figure 23:**
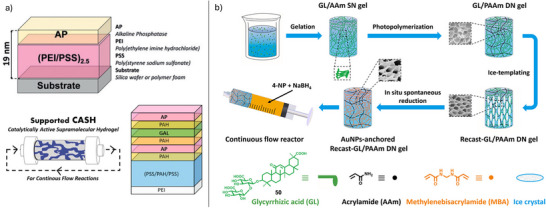
a) (Top) Multilayer system produced in an open cell melamine foam incorporating alkaline phosphatase (AP) enzyme that triggers the hydrolysis of Fmoc‐GFF*p*YGH*p*Y (not shown) to assemble a supramolecular gel. (Bottom Left) The resulting hybrid material constitutes a catalytically‐active supramolecular hydrogel (CASH) that can be used for continuous flow synthesis. (Bottom Right) Multilayer device incorporating both AP and galactosidase (GAL) enzymes. Figure adapted from References [83] and [156] with permission from Wiley‐VCH. b) Hybrid gel based on LMWG **50** and photopolymerised acrylamide PG is tough and stretchable. Growing ice crsytals within the gel increases porosity and surface area. The hybrid gel achieves in situ reduction of Au(III) to AuNPs and the AuNP‐loaded material was used to catalyse the reduction of 4‐nitrophenol. Figure reproduced from Reference [157] with permission from Wiley‐VCH.

As noted by Fores and coworkers, however, for the most desirable robust automated flow chemistry, supramolecular gels can struggle as a result of rheological weakness.^[^ ^83^ ^]^ Their approach to solving this problem was to combine supramolecular gels with other polymeric materials to harness synergistic performance. Specifically, they self‐assembled a peptide‐based gelator with esterase‐type activity within an open cell polymer foam.^[^ ^83^ ^]^ They used a commercial open‐cell melamine foam with cell diameters of about 200 µm, packed inside a metallic column. The walls of the pores were coated with a multilayer consisting of poly(ethyleneimine) (PEI), poly(styrenesulfonate) (PSS), and alkaline phosphatase (AP) by dipping alternately in PEI, PSS, and AP solutions (Figure 23a, top). Fmoc‐GFF*p*YGH*p*Y solution was then passed through the functionalized column, with the AP enzyme triggering gel assembly by phosphate hydrolysis—a perpendicular orientation of the self‐assembled gel fibers from the surface being observed. The catalytic activity of the assembled LMWG, which is an active hydrolysis catalyst, was first evaluated in an automated flow reactor using activated 4‐nitrophenyl acetate as substrate (1 mm) with a flow rate of 1.5 mL min^−1^. After 2.8 min in a closed loop, conversion was 98%. Interestingly, the researchers also noted that the reactor was highly effective in the hydrolysis of a range of inactivated esters—less often reported for this type of peptide LMWG. Further, their catalytically active supramolecular hydrogel (CASH) flow system (Figure 23a, bottom left) also achieved the isolation of quantitative amounts of enantiopure carboxylic acids from racemic or enantioenriched inactivated esters. The scope exemplified here, moving beyond ‘easy‘ reactions with activated substrates, will be important when creating next‐generation versatile synthetic tools based on gels. Importantly, there was no apparent loss of the peptide gelator from the column. The authors noted that continuous flow chemistry appears to be well suited for supramolecular gels as the flow can help compensate for the lower diffusion rate of substrates under static conditions.

The same workers also produced hybrid materials using simpler peptide LMWGs based on Nap‐FFY. In this case, the alkaline phosphatase was not only triggering gel assembly but was also then the source of catalytic activity, decorating the self‐assembled gel fibres and converting 4‐nitrophenylphospate into 4‐nitrophenol in continuous flow mode more effectively than the bare substrate.^[^ ^156^ ^]^ The authors fabricated another system that also incorporated β‐galactosidase as a second enzyme within the multilayer assembly process (Figure 23a, bottom right) and demonstrated that both enzymes were active, with the self‐assembled gel again improving performance compared with the bare film. Finally, the authors combined their esterase‐like peptide gel^[^ ^83^ ^]^ with this approach, and created a system which could catalyse the hydrolysis of both the phosphate group and the methyl ester group of Fmoc‐*p*Y‐OMe as a result of having both the AP and the peptide hydrogel present. The relatively easy fabrication of these multilayer flow‐through devices provides significant tunability and the ability to catalyse multistep reactions in a single device has considerable potential for future application.

Hao and coworkers have also taken a hybrid gel approach to creating robust flow reactors with supramolecular gels (Figure 23b).^[^ ^157^ ^]^ They assembled a supramolecular gel based on glycyrrhizic acid (GA, **50**) as an LMWG in acrylamide, with photo‐polymerisation then being used to cross‐link the polymer gel network. These hybrid LMWG/PG materials were tough and stretchable as a result of the polymeric component and subsequently had their porosity and surface area increased by using an ice‐templating method in which ice crystals were grown through the gel. The modified gel was used for in situ reduction of Au(III) to AuNPs as a result of the oxidation of the diglucuronic moiety of GA. As such, the carefully chosen LMWG also plays an active chemical role in this hybrid gel. The resulting AuNP‐loaded hybrid gel was used in an automated continuous flow reactor to catalyze reduction of 4‐nitrophenol to 4‐aminophenol, a process with quantitatve conversion that could be repeated for multiple (>20) cycles with no obvious decrease in activity.

As flow chemistry becomes increasingly embedded in synthetic chemistry laboratories, it seems likely that supramolecular gels, given their excellent solvent compatibility and porous networks, will play a role in developing systems capable of specific transformations. Gel properties will likely be optimized for this application using multicomponent formulation approaches as outlined in some of the examples above. Given the importance of mass transport in effective catalytic processes, it seems likely that in addition to the creation of bulk materials for use in automated synthesis, combining supramolecular gels with membranes will play an important role in enhancing the strength of such materials whilst maximising effective flow rates. As yet, the incorporation of LMWGs into membrane technology remains relatively unexplored. Combination of gels in flow with automation and continuous flow systems may allow such systems to be commercialized and ultimately become standardized tools for synthetic chemistry. Once again, the potential of spatial resolution within gel‐phase materials to generate materials with different reactivities opens the possibility of such devices being used to perform multistep reaction processes, adding very significant value compared with more typical synthetic workflows, and becoming powerful tools in the hands of synthetic chemists.

### 6.4 Patterning/Structuring Supramolecular Gels for Catalysis

There is the potential to apply a wide range of different physical gel patterning methods to achieve desired reaction outcomes. Indeed, the patterning of supramolecular gels is a field of intense current interest, with strategies ranging from 3D printing to diffusion patterning.^[^ ^84^ ^]^ Such methods are only just beginning to be applied to the wider field of reaction engineering. As such, work with supramolecular gels in this area is nascent, and there is great potential for future development.

**Figure 24:**
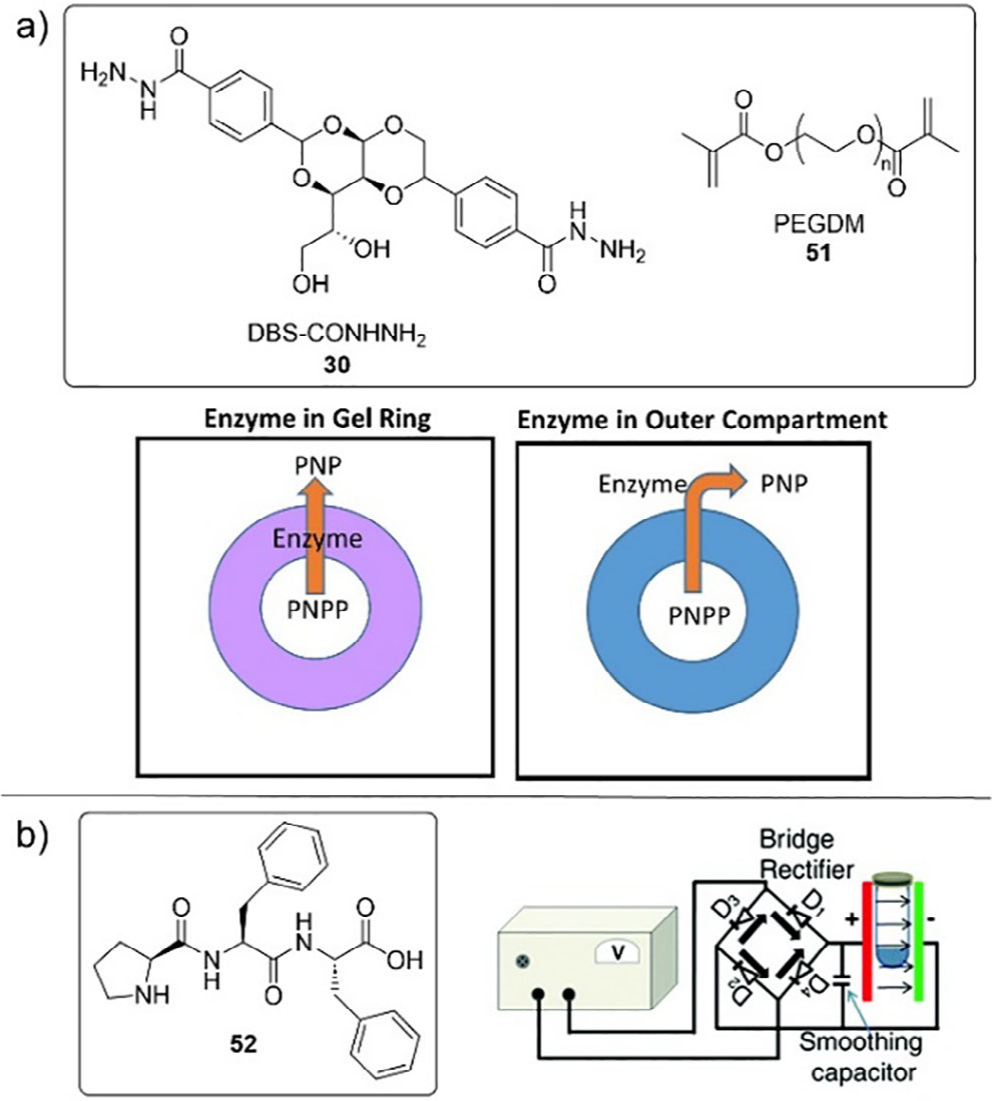
a) Design of ring‐shaped reactors based on the combination of LMWG **30** and PEGDM PG **51**, enabled flow‐through hydrolysis of 4‐nitrophenylphosphate (PNPP), driven by diffusion and concentration gradients. Figure adapted from Reference [158] with permission from the Royal Society of Chemistry. b) Schematic representation of the electric field set‐up with adjustable field strengths from 0 to 375 V cm^−1^ used for the modification of LMWG **52** assembly, with the resulting materials being used in catalysis of the aldol reaction. Figure adapted from Reference [159] with permission from the Royal Society of Chemistry.

In early preliminary work, we applied a photo‐patterning approach to reactive self‐assembled gels. In addition to thinking about enzyme‐loaded gels as a simple catalyst that can be added to a reaction system, in this way we demonstrated that it is also possible to consider using gels to shape ‘reactors’ that include enzymes. In such systems, the gel plays a different type of active role, controlling the diffusion of reagents and products through the gel in order to ensure contact with the catalytically active unit in a spatially controlled manner. With the ultimate goal of creating shaped gel “reactors” that could be driven by simple diffusion or active pumping, we created first‐generation ring‐shaped gels with encapsulated phosphatase enzymes (Figure 24a).^[^ ^158^ ^]^ To achieve this, LMWG DBS‐CONHNH_2_ (**30**) and photopolymerizable poly(ethylene glycol dimethacrylate) (PEGDM, **51**), along with a photoinitiator, were combined and the LMWG then allowed to assemble in a square tray. Photoirradiation under a ring‐shaped mask led to polymerization of PEGDM, giving a very robust hybrid PG/LMWG ring‐shaped gel. The surrounding soft LMWG‐only matrix could be simply washed away with spraying from a water bottle. This fabrication approach, therefore, harnesses the reversibility of the LMWG matrix to actually enable hybrid gel shaping. The preformed LMWG matrix helps enforce the patterning of the PG ring with excellent spatial resolution by limiting diffusion and convection during photoirradiation, while could also be simply removed as a result of its rheological weakness. Phosphatase enzymes were either incorporated in the gel ring prior to its fabrication or were placed in the outer solution‐phase compartment after fabrication was complete. On loading 4‐nitrophenylphosphate (PNPP) into the interior solution‐phase compartment, diffusion through the reactor ring led to products being released into the outer compartment (or only formed once diffusion through the ring had taken place). It is possible to imagine creating smart catalytically active gels for pumped flow chemistry using this type of approach.

As an alternative to using light to manipulate gels, Ramakrishnan and coworkers elegantly demonstrated that an external physical stimulus in the form of an electric field can potentially be combined with self‐assembly to enhance catalysis.^[^ ^159^ ^]^ Specifically, they applied an electric field during the cooling and self‐assembly of tripeptide **52** based on proline and phenylalanine (Figure 24b). On removing the gel from the electric field and testing it in the catalysis of the aldol reaction between cyclohexanone and 4‐nitrobenzaldehyde, they found the gel that had been exposed to a 300 V cm^−1^ electric field had its enantioselectivity enhanced by 36% (from 53% ee to 89% ee). At lower electric fields (150 V cm^−1^), the ee was intermediate at 66%. The authors observed some changes in the circular dichroism spectrum of the self‐assembled gel on application of an electric field, and thus suggested the π‐electrons of the LMWG may make it susceptible to polarization effects that may affect assembly. Although the direct mechanism by which this impacts catalysis could not be unpicked, this example elegantly demonstrates how gel “engineering” as well as just simple self‐assembly can direct reaction outputs and the way in which physical inputs can be used to tune catalytic performance.

It seems likely, as the use of LMWGs in synthetic chemistry moves from being of primarily supramolecular interest to becoming a laboratory‐based technology with real‐world applications, that engineering methods to manipulate and optimize the performance of gel‐phase materials will become increasingly important. Understanding how gels respond to a variety of physical inputs, and ideally in a predictive sense how these impact on catalytic outputs, will be of considerable future importance.

## 7 Conclusions and Prospects

The unique properties and characteristics of both the nanoscale solid‐like phase and the bulk liquid‐like medium of supramolecular gels can give valuable synthetic outcomes. In terms of the solid‐like phase, the organization of molecular‐scale building blocks can yield enhanced catalytic performance and modify reaction selectivity. A number of key principles have emerged such as the ability of gel assembly to modify p*K*a values, change sterics or electronics of the local environment at the catalytic site, or provide hydrophobic domains capable of enhancing reactivity of hydrophobic substrates. It is worth noting that results in this area are often compared against the same reactions carried out in solution‐phase conditions. In future, there may be more need to benchmark such systems against other supported catalysts in order to gain more detailed insight into the differences between different types of supported material and the unique potential advantages of gels. Ideally, this type of understanding would eventually help unlock a greater degree of ab initio prediction the way in which an LMWG would assemble, the nanostructures it would form, and as a result, the impact the assembled LMWG would have on reaction processes. At present, the state‐of‐the art is to try and rationalize these factors, but if computational methods enabled a greater degree of design, this would radically transform the way the field develops, moving from trial‐and‐error discovery to rational targeted design of gel‐based synthetic tools.

With increasing interest in the way chemistry led to the evolution of life, it is likely that organocatalytic work using gels will increasingly focus on reactions of potential importance on the early Earth and will also attempt to make protocell type materials that make use of minimalistic building blocks yet achieve interesting emergent spatially localised functions based on controlled chemical reactivity.

Loading biocatalysts into supramolecular gels is an effective way of creating catalytic gels. In some cases, the encapsulated enzyme exhibits superactivity. Furthermore, the gel network can enhance enzyme stability and prevent denaturing, which offers genuine future possibility for the use of gels where bioactive agents have to be stored, transported, or used in challenging conditions. Beyond encapsulating whole enzymes, it is possible to use gel networks to encapsulate bioactive subunits, with the organization imposed by the gel environment leading them to cooperate—such systems can be considered “nanozymes”. There is a particular interest in biomimetic gels that generate reactive oxygen species. Clearly, there is an interface here with work on gel‐based protocells. It is suggested that future work will target the development of gels that can act as complex bio‐nanoreactors, capable of carrying out multiple orthogonal processes in a manner increasingly reminiscent of living systems.

The stabilizing effect of the gel microenvironment can extend to other reactive species enabling their easy use in ambient conditions. For example, gels have been used to stabilize metal nanoparticles including ligand‐free systems that would otherwise be unstable toward aggregation. Eye‐catchingly, organogels have been used to stabilize highly reactive species such as organolithiums, organomagnesiums, and organoboranes. These gels show remarkable stability under ambient conditions, can be handled easily in the laboratory, and dosed into reactions with ease. This opens the possibility of transforming the way such challenging reagents are used in synthetic chemistry, opening up their potential to a much wider range of nonspecialist users. It also suggests transformative potential for the transport, storage, and bulk use of such reagents.

Even in the absence of specific catalytic or reactive features in the LMWG or additives within the gel network, there is considerable potential for the use of gels as “nanoreactors”. The interior microenvironment often has unique features, such as chirality, confined self‐assembled nanoenvironments, coexisting hydrophobic and hydrophilic domains, or functional groups that may bind to reagents. Such aspects can promote a range of reactions, and potentially lead to unusual outcomes. As of yet, these aspects of gels in synthesis are somewhat under‐explored, and there may be significant advances in this area to develop some ground rules for gel nanoreactor design.

When applying gels in synthetic chemistry, there are a wide range of ways in which the material can be engineered to enable easy workflows that harness some of their unique advantages. For example, gels can be fabricated as capsules or beads that can be easily manipulated by end‐users. This can be facilitated by high LMWG loadings or coformulation with additives that provide rheological strength. Indeed, coformulation or coassembly approaches have a great deal of power in terms of formulating gels with multiple different properties. Ultimately, it should be possible to fabricate gels that can perform multistep reaction processes as a result of their design, for example, layer‐by‐layer materials that release/expose reactive reagents sequentially into a reaction system. In the development of such systems, as for all gel‐based catalysts, it will be important to ensure robust reusability of the materials in order to maximize green and sustainable aspects of synthetic workflows—in published work, this aspect is only sporadically investigated.

Gels are ideally suited to flow chemistry as a result of their highly solvated nature. Although flow chemistry with supramolecular gels has most often been performed in simple gravity‐driven devices, in some cases, gels have been developed into automated continuous flow systems. In general, however, the application of supramolecular gels in commercial flow systems remains at a very early stage, and there is considerable potential for these materials to help develop “kits” with different reactivity profiles, which could simply be inserted into commercial flow systems and used in synthetic chemistry. It is suggested that in the future, the combination of supramolecular gels with membrane technology may enable effective combination of gel performance with stable rheological performance and effective mass transport. Once again, the potential for gels to mediate multistep reactions in flow, as a result of the capacity to load orthogonal reagents within them, has particular promise in terms of telescoping and simplifying the way in which complex molecules are made.

Finally, the ability to fabricate microscale gel particles with unique, orthogonal, reactive profiles, opens the possibility of creating unique reactive species that can potentially be manipulated, rather like cellular objects, to intervene in a range of processes with spatial resolution or creating life‐like types of behavior. It is expected that there will be significant developments in this area over the coming years, potentially finding an interface between the types of synthesis done within biological systems, and what can be achieved using a more traditional synthetic chemistry approach.

In summary, research at the interface between synthesis and supramolecular gels is an exciting frontier. Supramolecular gels not only have the potential to change the ways in which synthesis is done but also to help move the understanding of synthetic chemistry beyond traditional flask‐based methodologies. Along the way, a detailed understanding of fundamental principles of supramolecular chemistry and soft materials engineering will be integral in maximizing the potential of these materials. It seems likely that advances in gels will not only lead to advances in synthesis but also that advances in synthesis using gels will lead to new concepts that will impact much more widely across different scientific fields.

## Conflict of Interests

As a conflict of interest, D.K.S. notes that he is one of the inventors on the patent protecting the organolithium gel technology (WO2021214453A1).

## Data Availability

Data sharing is not applicable to this article as no new data were created or analyzed in this study.
